# Human loss-of-function variants in the serotonin 2C receptor associated with obesity and maladaptive behavior

**DOI:** 10.1038/s41591-022-02106-5

**Published:** 2022-12-19

**Authors:** Yang He, Bas Brouwers, Hesong Liu, Hailan Liu, Katherine Lawler, Edson Mendes de Oliveira, Dong-Kee Lee, Yongjie Yang, Aaron R. Cox, Julia M. Keogh, Elana Henning, Rebecca Bounds, Aliki Perdikari, Vikram Ayinampudi, Chunmei Wang, Meng Yu, Longlong Tu, Nan Zhang, Na Yin, Junying Han, Nikolas A. Scarcelli, Zili Yan, Kristine M. Conde, Camille Potts, Jonathan C. Bean, Mengjie Wang, Sean M. Hartig, Lan Liao, Jianming Xu, Inês Barroso, Jacek Mokrosinski, Yong Xu, I. Sadaf Farooqi

**Affiliations:** 1 USDA/ARS Children’s Nutrition Research Center, Department of Pediatrics, Baylor College of Medicine, Houston, TX USA; 2 University of Cambridge Metabolic Research Laboratories and NIHR Cambridge Biomedical Research Centre, Wellcome-MRC Institute of Metabolic Science, Addenbrooke’s Hospital, Cambridge, UK; 3 Department of Molecular and Cellular Biology, Baylor College of Medicine, Houston, TX USA; 4 Division of Diabetes, Endocrinology and Metabolism, Department of Medicine, Baylor College of Medicine, Houston, TX USA; 5 Exeter Centre of Excellence for Diabetes Research (EXCEED), University of Exeter Medical School, Exeter, UK

**Keywords:** Metabolism, Behavioural genetics, Molecular neuroscience

## Abstract

Serotonin reuptake inhibitors and receptor agonists are used to treat obesity, anxiety and depression. Here we studied the role of the serotonin 2C receptor (5-HT_2C_R) in weight regulation and behavior. Using exome sequencing of 2,548 people with severe obesity and 1,117 control individuals without obesity, we identified 13 rare variants in the gene encoding 5-HT_2C_R (*HTR2C*) in 19 unrelated people (3 males and 16 females). Eleven variants caused a loss of function in HEK293 cells. All people who carried variants had hyperphagia and some degree of maladaptive behavior. Knock-in male mice harboring a human loss-of-function *HTR2C* variant developed obesity and reduced social exploratory behavior; female mice heterozygous for the same variant showed similar deficits with reduced severity. Using the 5-HT_2C_R agonist lorcaserin, we found that depolarization of appetite-suppressing proopiomelanocortin neurons was impaired in knock-in mice. In conclusion, we demonstrate that 5-HT_2C_R is involved in the regulation of human appetite, weight and behavior. Our findings suggest that melanocortin receptor agonists might be effective in treating severe obesity in individuals carrying *HTR2C* variants. We suggest that *HTR2C* should be included in diagnostic gene panels for severe childhood-onset obesity.

## Abstract (web summary)

Identification and characterization of variants in *HTR2C* in people with severe obesity, reveals a role for serotonin 2C receptor in regulation of appetite, weight and behavior.

## Main

Drugs that alter levels of the neurotransmitter serotonin (5-hydroxytryptamine, 5-HT) are widely prescribed for the treatment of obesity and neuropsychiatric disorders; however, they often exert adverse effects due to a lack of receptor specificity^1^ as 5-HT signals through at least 14 different receptors to regulate body weight, mood and behavior^2,3^. For example, second-generation antipsychotic drugs (clozapine and olanzapine) are highly effective at reducing psychotic symptoms, but cause increased hunger and weight gain in up to 60% of patients, which represents a major barrier to their long-term use^4^. Understanding the mechanisms by which serotonin’s effects on food intake, body weight, mood and behavior are mediated in humans could inform the development of more targeted therapies for a range of clinical disorders.

Studies in mice have shown that the appetite-suppressing actions of 5-HT are largely mediated by 5-HT_2C_Rs expressed on hypothalamic proopiomelanocortin (POMC) neurons^5–7^, which play a major role in weight regulation. A complete lack of POMC due to bi-allelic loss-of-function mutations causes hyperphagia and severe childhood-onset obesity^8^. This genetic obesity syndrome and other closely related disorders of the leptin–melanocortin pathway are treatable by setmelanotide, a melanocortin 4 receptor (MC4R) agonist, which has been licensed for clinical use in the UK, Europe and the US^9–11^. Here, we set out to investigate the potential contribution of 5-HT_2C_R signaling to human weight regulation and the interaction between 5-HT_2C_Rs and the melanocortin pathway.

## Results

### Rare variants in *HTR2C* in people with severe obesity

**Fig. 1:**
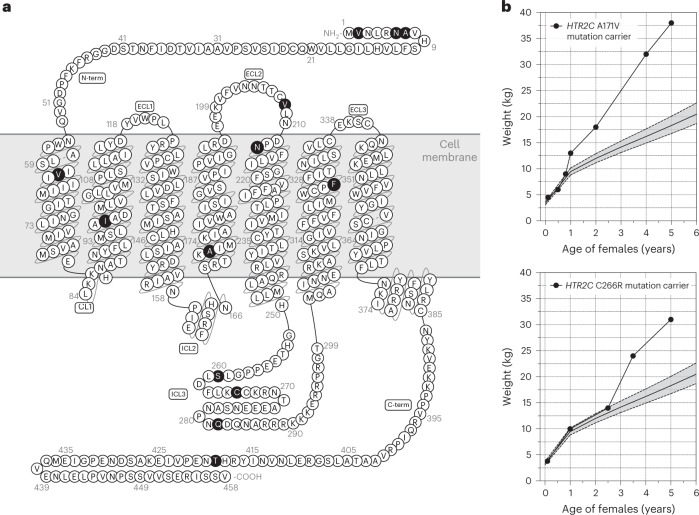
Rare variants affecting 5-HT_2C_R identified in people with severe obesity. **a**, Rare variants identified in individuals with severe early-onset obesity shown on a schematic of the 5-HT_2C_R protein; ECL and ICL refer to extra- and intracellular loops of the G-protein-coupled receptor (GPCR), respectively; C-term, C-terminal domain of the protein. **b**, Weight charts of two female probands (5th and 95th percentiles based on reference data for the UK population shown as dashed lines).

We analyzed exome sequencing and targeted resequencing on 2,548 (46% male and 54% female) European ancestry individuals with severe, early-onset obesity (mean body mass index (BMI) s.d. score (SDS) > 3; age of onset <10 years) and 1,117 (40% male, 60% female) ancestry-matched controls analyzed using the same methods^12,13^. We identified 13 rare variants (minor allele frequency <1%) in *HTR2C* in 19 unrelated people with severe obesity (Fig. 1a); these variants were either not found or very rarely found, in publicly available exomes (Table 1). One rare variant (A171V) was identified in obese individuals (*n* = 5) and controls (n = 2); no rare variants were found in controls only. *HTR2C* lies on the X chromosome in humans; 16 girls carried a heterozygous variant and 3 boys carried a hemizygous variant (variant on their only X chromosome). Three families of probands carrying variants that were not found in controls, consented to co-segregation studies. The four people carrying rare *HTR2C* variants in these families had overweight or obesity (Table 1).

**Table 1:** Phenotypes seen in people carrying rare variants in *HTR2C*

Variant	Age	Sex	Weight	Height	BMI	Measured BMR	Predicted BMR	RQ	Glucose	Insulin	SBP/DBP	Leptin	TSH	FT4	Medical history
	(years)		kg	cm (SDS)	kg m^−2^ (SDS)	MJ d^−1^	MJ d^−1^		mmol l^−1^	pmol l^−1^	mm Hg	nmol l^−1^	IU l^−1^	IU l^−1^	
V2L*	27.3	F	161.2	154.6	67.4	9.3	10.5	0.9	4.7		123/59		2.9	13.2	Exomphthalos, mild learning difficulties, sleep apnea
N6K*	5.6	F	40.1	119.2 (1.4)	28.2 (4.3)				4.4	41		31.1			
A7V*	19.3	F	107.6	162.5	40.8	7.5	7.9	0.9	4.8	130	116/70		3.8		Dyslexia, learning difficulties, emotional lability, volatile behavior, delayed puberty
A7V (m)	44	F	75.5	165.1	27.7										
A7V*	13.4	F	101	154.0 (−0.5)	42.6 (3.9)										
V61I*	13.5	F	102	158.3 (0.1)	40.7 (3.8)				Normal	-					ADHD, social anxiety, depression, ODD, borderline personality disorder
V61I*	19	F	114.4	155.3	47.4	8.5	7.4	0.7	4.6	212	105/59		1.4	12.1	Dyslexia, learning difficulties, volatile behavior, anxiety, aggression, depression, emotional lability, impulsivity, mild OCD and social anxiety
V61I (m)	43.9	F	89.1	160.0	34.8										Depression, anxiety
V61I (s)	25.4	F	102.2	159.0	40.4	7.5	7.7	0.8	4.3	95	134/85		0.7	11.7	Anxiety, asthma
I97V*	11	F	82	157.0 (1.9)	33.3 (3.4)				IFG	431			Normal	Normal	
A171V*	14.6	M	108.5	172.2 (0.7)	36.6 (3.4)	9.8	9.2		5.4	265	110/63	89	2.2	16.4	
A171V (m)	39.6	F	64.1	173.8	21.2	6.4	5.9	0.9	5.8	68	124/83	35.3	0.7	17	
A171V (b)	9.9	M	31.2	142.2 (0.7)	15.4 (-0.6)	4.5	4.7	0.9	4.7	94	99/64	19.8	2.2	14.6	
A171V(mgf)	71.3	M	86.7	176.1	28	7.6	7.9	0.9	5.1	44	127/73		2.0	15.7	
A171V(ma)	43.1	F	76.4	167.6	27.2										
A171V*	5.1	F	29.7	110.2 (0.1)	24.5 (3.7)				4.7	202		39.1			Learning difficulties, emotional lability. Facial dysmorphia
A171V*	1.2	M	15.2	83.5 (2.0)	21.8 (2.7)				Normal	-					
A171V*	2.6	F	20.5	84.0 (−2.1)	29.1 (5.4)				Normal	-			5.0	16.6	Learning difficulties, severe delay. Slight facial dysmorphia, increased infections
A171V*	7	F	34.7	116.5 (−0.9)	25.6 (3.3)				4.5	43			Normal	Normal	Speech delay, attention deficit disorder, hyperactivity
V208M*	3.1	F	16.5	85.7 (−2.6)	22.5 (3.5)				-	-			Normal	Normal	Learning disability
N213T*	6.4	M	35.9	122.0 (0.8)	24.1 (3.6)				5.2	72		16.7	Normal	Normal	Epilepsy
S260G*	16.1	F	127	154.0 (−1.5)	53.6 (4.4)				Type 2 diabetes	559		114			Depression, social anxiety, PCOS
C266R*	15.8	F	112	156.4 (−1.1)	45.8 (4.0)	6.8	7.9	0.9	3.8	251	113/68		3.3	14.6	Learning difficulties, delayed speech, autistic traits, social anxiety, depression, aggression, emotional lability, behavioral & concentration difficulties
C266R (s)	33.9	F	171.8	170.2	59.3										Aggression
C266R (m)	56.1	F	104.6	165.1	38.4						Hypertension				
Q282H*	15.9	F	82.7	156.0 (−1.2)	34.0 (3.0)				3.9	62		23.1			Depression, anxiety, sudden mood swings and PCOS
F327L*	7	F	49.8	126.4 (1.0)	31.2 (4.2)				6.3	103		44	Normal	Normal	Learning difficulties, volatile behavior and PCOS
F327L (f)	30.9	M	88.9	1.78	28.1										
Tf419A*	7.1	F	40.8	128.3 (1.3)	24.8 (3.1)				Normal					Normal	Hypothyroidism, social anxiety

Data for probands with severe obesity (denoted as *) from the GOOS cohort in whom *HTR2C* variants were identified. Variants were found in hemizygous form in males and heterozygous form in females (transcript ID, ENST00000276198). Additional information was obtained on their family members who carried variants, which is included directly beneath the proband where available; family relationships are indicated in brackets (m, mother; f, father; ma, maternal aunt; s, sister; b, brother; mgf, maternal grandfather.) Age and sex-adjusted SDS for height and BMI were included for individuals up to 18 years; sex (M, male; F, female); TSH, thyroid stimulating hormone (0.35–5.50 mIU l^−1^); FT, free thyroxine (10.0–19.8 pmol l^−1^); fasting plasma insulin (0–60 pmol l^−1^); fasting glucose (3.5–5.5 mmol l^−1^). In some cases, biochemical test results were not available but values were recorded as within the normal range in clinical letters (recorded as normal here). Basal metabolic rate (BMR) and respiratory quotient (RQ) were measured by indirect calorimetry in the fasted state and BMR was predicted on the basis of age, sex and body composition shown for comparison. Systolic blood pressure (SBP) and diastolic blood pressure (DBP) were measured in the rested, fasted state in mm Hg. Blank indicates data not available. ADHD, attention deficit hyperactivity disorder; ODD, oppositional defiant disorder; OCD, obsessive-compulsive disorder; IFG, impaired fasting glycemia; PCOS, polycystic ovarian syndrome.

### Clinical phenotype of carriers of rare variants in *HTR2C*

All probands had a history of hyperphagia, impaired satiety and weight gain from early childhood (Fig. 1b) leading to severe obesity (mean BMI = 36.5 kg m^−2^ in adults; mean BMI SDS = 3.5 in children) (Table 1). Eight of 19 probands had learning difficulties or developmental delay; longitudinal follow-up of patients revealed a wide spectrum of maladaptive behaviors that started in childhood, including emotional lability (frequent outbursts of crying and/or aggressive behavior in the absence of obvious triggers) and maladaptive behavior, particularly in social settings (Table 1).

**Fig. 2:**
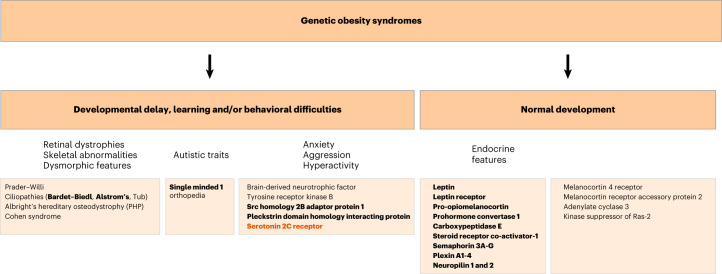
Genetic obesity syndromes. A schematic depicting genetic obesity syndromes, conditions where severe childhood-onset obesity is a major presenting clinical feature. Conditions in bold are those where the gene/genes have been shown to affect signaling through the leptin–melanocortin pathway and could be treated by an MC4R agonist. Clinical features seen in people with LOF variants in the gene encoding the serotonin 2C receptor (orange) are reported in this study.

The clinical features seen in people carrying *HTR2C* variants show some overlap with other conditions associated with onset of severe obesity in childhood (Fig. 2). For example, reactive aggression is seen in people with deletions and loss-of-function (LOF) mutations in the adaptor molecule, *SH2B1*, which modulates signaling by leptin, insulin and brain-derived neurotrophic factor (BDNF)^14,15^. In our study, people carrying *HTR2C* variants generally reported features of anxiety and/or social anxiety from childhood. To ascertain the prevalence of these conditions in a large clinically ascertained cohort, we reviewed the records of 7,775 children recruited to the Genetics of Obesity Study (GOOS; www.goos.org.uk). Criteria for inclusion in this study were severe early-onset obesity (defined as BMI SDS > 3, onset of obesity before 10 years of age). Learning difficulties (*n* = 650, 8.3%), speech and language delay (*n* = 609, 7.8%) and autism spectrum disorder (*n* = 382, 4.9%) were frequently reported at the time of recruitment (mean age 10 years). Behavioral issues (not solely around food) were reported in 271 children (3.4%), hyperactivity (*n* = 112, 1.4%) and anxiety or social anxiety (*n* = 77, 1%) were sometimes reported.

**Extended Data Fig. 1:**
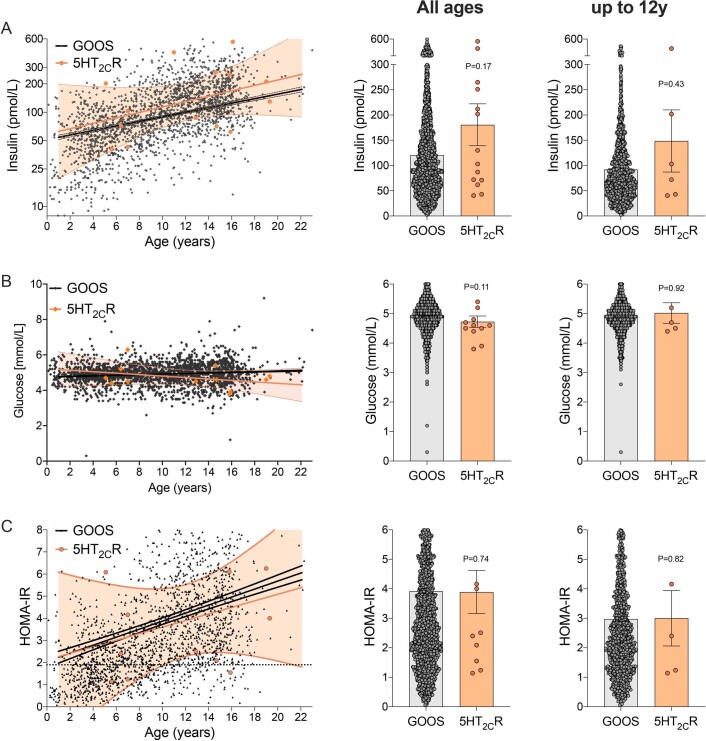
Glucose homeostasis in *HTR2C* variant carriers compared to age and BMI matched controls. **a**, Fasting plasma insulin levels (log_10_-transformed) in 14 *HTR2C* variant carriers compared to 2138 severely obese children as controls [GOOS]. **b** and **c** show the mean fasting plasma insulin levels in *HTR2C* variant carriers of all ages (N = 14) or under 12 years of age (N = 6). **d**, Fasting plasma glucose levels in 12 *HTR2C* variant carriers compared to 2248 severely obese children as controls [GOOS]. **e** and **f** show the mean fasting plasma glucose levels in *HTR2C* variant carriers of all ages (N = 12); and in *HTR2C* variant carriers up to 12 years of age (N = 5), respectively. In **a** and **d**, the 5^th^ and 95^th^ percentiles are shown as dashed lines and the regression slopes are shown as solid lines in gray [GOOS] or orange [*HTR2C* variant carriers]. **g**, The relationship between HOMA-IR (Homeostasis Model Assessment of insulin resistance) scores and age in 12 *HTR2C* variant carriers compared to 2024 severely obese children as controls [GOOS]. HOMA-IR was calculated as [(fasting insulin in μU/mL) × (fasting glucose in mg/dL)] / 405. Dashed lines represent 95% confidence intervals; regression slope shown as solid lines in gray [GOOS] or orange [*HTR2C* variant carriers]. A dotted horizontal line indicates the threshold above which values are consistent with insulin resistance. **h** and **i** show the mean HOMA-IR scores in *HTR2C* variant carriers of all ages (N = 12); and on *HTR2C* variant carriers up to 12 years of age (N = 5), respectively. Statistical significance was determined using two-tailed Mann-Whitney U test. Data are shown as mean ± SEM.

To investigate the clinical phenotypes seen in people carrying *HTR2C* variants, we invited all probands and their family members to take part in physiological studies (Methods); six individuals agreed to take part. Body composition measured by dual energy X-ray absorptiometry showed excess body fat mass (mean (± s.d.) percentage body fat 46.6 ± 14.5) and a normal bone mineral density (BMD) SDS (mean 0.38). Basal metabolic rate measured by indirect calorimetry after an overnight fast was comparable to that predicted on the basis of age, sex and body composition (Table 1). Carriers of variants had normal blood pressures measured in the rested, fasted state (Table 1). Fasting glucose and insulin levels and HOMA-IR (homeostasis model assessment of insulin resistance) were appropriate for the degree of obesity when people carrying variants were compared to 2,138 controls of a comparable age, sex and BMI (Extended Data Fig. 1).

### Functional characterization of *HTR2C* variants

To investigate the functional consequences of rare variants in *HTR2C*, we performed a series of experiments in cells transiently transfected with constructs encoding wild-type or mutant human 5-HT_2C_R. The functional activity of Gα_q/11_-coupled 5-HT_2C_Rs is regulated by desensitization and re-sensitization which involves the recruitment of intracellular β-arrestins that promote the phosphorylation of extracellular signal-regulated kinase 1/2 (ERK1/2)^16,17^ and thus, gene transcription. The site where 5-HT_2C_R interacts with G proteins is prone to RNA editing, which affects the receptor’s affinity for G proteins and hence its constitutive activity^18,19^. We studied variants in the ‘VSV’ isoform, the most prevalent isoform in the human hypothalamus^20^.

**Fig. 3:**
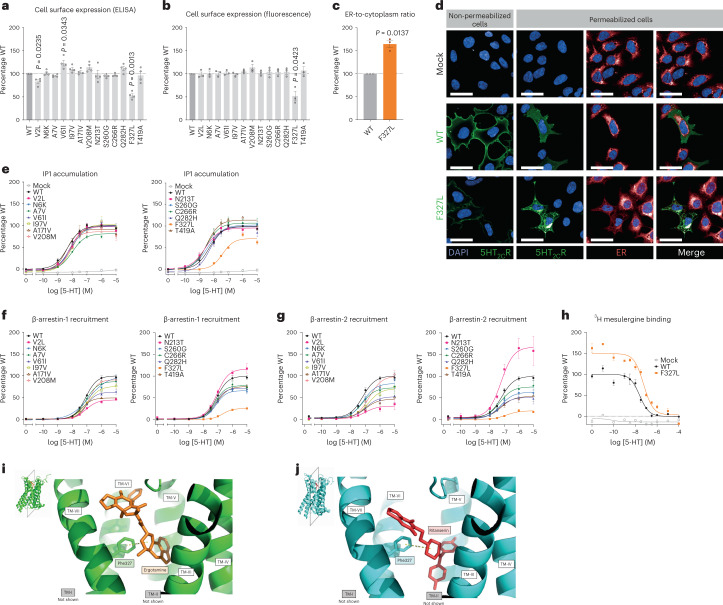
Human variants in the gene encoding 5-HT_2C_R cause a loss of function in cells. Wild-type (WT) and mutant forms of 5-HT_2C_R were studied in cells. **a**,**b**, Cell surface localization of WT and mutant receptors measured by ELISA (**a**) and high-content confocal microscopy (**b**). Values are expressed as percentage of WT and represent mean ± s.e.m., *n* = 3–4 independent experiments; mock transfected cells served as negative controls. Differences between WT and mutant receptors were compared using two-tailed Student’s *t*-tests with Welch correction. **c**, Expression of WT versus F327L 5-HT_2C_R in the ER compared to the cytoplasm, quantified by high-content confocal microscopy. Data represent mean ± s.e.m., *n* = 3 independent experiments; differences between WT and mutant receptors were determined by two-tailed Student’s *t*-tests with Welch correction. **d**, A representative micrograph of subcellular localization of WT and F327L 5-HT_2C_R in non-permeabilized and permeabilized cells. Scale bars, 50 μm. 4,6-Diamidino-2-phenylindole (DAPI) was used to stain the nucleus. **e**–**g**, Effects on receptor-mediated activation of Gα_q/11_-regulated inositol triphosphate signaling (IP_1_ accumulation; *n* = 3–4 independent experiments) (**e**) and coupling to β-arrestin-1 and -2 (*n* = 4–5 independent experiments) (**f**,**g**). Data are shown as sum curves normalized to WT response ± s.e.m. EC_50_ and maximum response (E_max_) for each variant is shown in Supplementary Table 1. **h**, Competitive binding assay for WT versus F327L 5-HT_2C_R using the receptor antagonist ^3^H-mesulergine with increasing concentrations of the agonist, 5-HT. Sum curves are shown from *n* = 3 independent experiments normalized to WT max binding ± s.e.m. Affinity (IC_50_) and number of binding sites (B_max_) are shown in Supplementary Table 1; significant differences from receptor were determined using a two-tailed Student’s *t*-test with Welch correction. **i**,**j**, Structural analysis of the 5-HT_2C_R in an active (**i**) and inactive conformation (**j**), bound to the agonist ergotamine and the inverse agonist ritanserin, respectively. TM, transmembrane domains (I–VII). The F327 (Phe327) residue is highlighted and predicted to interact with both ligands^21^. Source data

**Extended Data Fig. 2:**
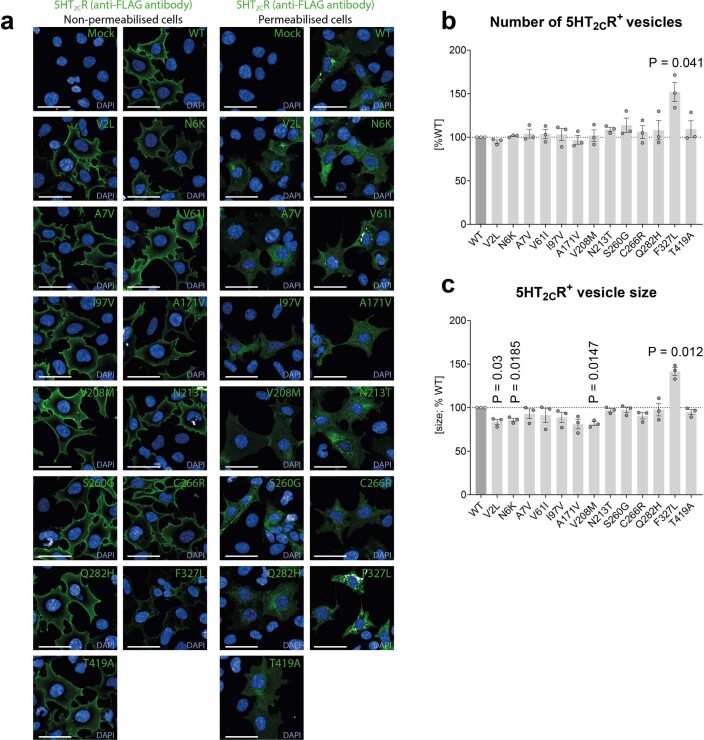
Functional characterization of human variants in 5-HT_2C_R. **a**, Representative micrographs of cell surface and intracellular expression of WT vs mutant receptors (green), quantified by high-content confocal microscopy. Phalloidin and DAPI (blue) were used to stain the cytoplasm and nuclei, respectively and were used for cell identification. Scale bar = 50 μm. **b**-**c**, Number of receptor-containing vesicles (**b**), and the size of these receptor positive vesicles (**c**), expressed as % of WT. Differences between WT and mutant receptors were compared with two-tailed student’s t-test with Welch correction. Results represent mean ± SEM of 3 independent experiments. Source data

Using a cell surface ELISA and high-content confocal microscopy to quantify receptors in different cellular compartments, we observed markedly reduced plasma membrane expression of one mutant, F327L (53% of wild-type, by ELISA; and 52% of wild-type, by microscopy) and slightly reduced expression of another (V2L) (83% of wild-type, by ELISA) (Fig. 3a,b, Extended Data Fig. 2a, and Supplementary Table 1). F327L 5-HT_2C_R colocalized with the endoplasmic reticulum (ER) marker, calreticulin (Fig. 3c,d and Extended Data Fig. 2b,c) consistent with its partial retention in this cellular compartment; other mutants did not affect cellular localization. We next quantified ligand-induced IP_3_ turnover and found that, compared to wild-type 5-HT_2C_R, F327L resulted in a LOF, decreasing both the maximal efficacy (E_max_) of signaling and increasing the half-maximal effective concentration (EC_50_) (Fig. 3e, Supplementary Table 1); none of the other mutants affected signaling in this assay. Using a NanoBiT protein–protein interaction assay, we found that nine mutants reduced agonist-induced β-arrestin-1 recruitment (Fig. 3f and Supplementary Table 1) and eight reduced β-arrestin-2 recruitment, (Fig. 3g and Supplementary Table 1); F327L increased the EC_50_ in assays for both β-arrestin-1 and -2 recruitment (Supplementary Table 1). In total, 11 of 13 mutants found in individuals with severe obesity caused a LOF in at least one functional assay. The variant found in people with obesity and controls (A171V) caused a LOF in HEK293 cells.

Peng and colleagues reported the crystal structure of 5-HT_2C_R bound to the agonist ergotamine and the inverse agonist ritanserin^21^. Their model predicted the F327 residue to be critical to binding of these compounds to the receptor. Moreover, substituting F327 to a leucine impaired predicted intermolecular (π–π) interactions and reduced the receptor’s affinity for ergotamine and 5-HT^21^. Here, in a competitive binding assay with the 5-HT_2C_R antagonist mesulergine, we observed decreased 5-HT binding affinity and higher maximal mesulergine binding (Fig. 3h), the latter suggesting that the F327L substitution stabilizes the inactive conformation of the receptor (Fig. 3i,j). Cumulatively, these studies demonstrate that the F327L variant causes severe LOF by disrupting a critical residue involved in the binding of 5-HT. Ten other missense mutations found in individuals with severe obesity caused a partial LOF, predominantly by affecting the recruitment of β-arrestins.

### Rare *HTR2C* variants in a population-based cohort

To investigate whether rare variants in *HTR2C* are associated with increased BMI or obesity in the population, we examined 200,000 exomes from a large population-derived cohort, the UK Biobank^22^. As *HTR2C* is located on the X chromosome, we performed analyses separately for unrelated males (*n* = 69,488) and females (*n* = 83,864) of European ancestry. Five people in the UK Biobank dataset had frameshift or stop-gain mutations in *HTR2C*; three of the five males had a BMI > 30 kg m^−2^; there were no female carriers. We studied rare *HTR2C* variants using gene-region and single-variant association analysis for obesity (BMI > 30 kg m^−2^), severe obesity (BMI > 40 kg m^−2^) and for BMI as a continuous trait (Methods and Supplementary Table 2). In males, gene-region rare variant tests showed a nominal association of rare (allele frequency <0.1%) non-synonymous exonic or splice-site variants and obesity (BMI > 30 kg m^−^^2^, *P* = 0.01, SKAT binary robust burden; *P* = 0.01, SKATO; odds ratio (OR) (95% CI) = 1.9 (1.1–3.1); *P* = 0.01, Fisher’s exact test). Among females, we found eight non-synonymous coding variants with an allele count >20 including three variants identified in the GOOS cohort (V61I, A171V and T419A); none was associated with BMI in single-variant analyses (Methods). Among variants identified in the GOOS cohort and shown to cause LOF in cells (allele count <10 in the UK Biobank), four of the five people carrying the Q282H *HTR2C* variant had obesity (BMI > 30 kg m^−2^; OR (95% CI) = 14 (1.3–670), nominal *P* = 0.01, Fisher’s exact test), 5 of 9 males carrying A7V had obesity (BMI > 30 kg m^−2^; *P* = 0.03, SKAT robust; OR (95% CI) = 3.8 (0.8–19), nominal *P* = 0.05, Fisher’s exact test).

We performed exploratory analysis of mental health data collected as part of the UK Biobank (*n* = 53,174; Methods and Supplementary Table 2). The V61I *HTR2C* variant was identified in 24 unrelated people in the UK Biobank; Mental Health Questionnaire data were available for ten of these individuals, five of whom (2 males and 3 females) had a diagnosis of depression; Fisher’s exact test, OR (95% CI) = 3.7 (0.8–16), *P* = 0.04 and three had additional diagnoses of ‘anxiety, nerves or generalized anxiety disorder’ or ‘phobias’; while individual associations do not survive multiple testing across traits and variants, these findings are compatible with our hypothesis that rare *HTR2C* variants contribute to obesity and maladaptive behavior in humans.

### Knock-in *Htr2c*^F327L^ mice develop hyperphagia and obesity

**Fig. 4:**
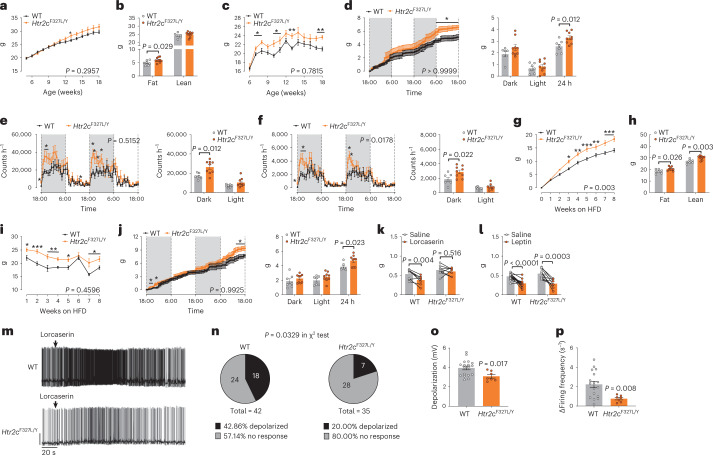
Knock-in *Htr2c*^F327L^ mice develop hyperphagic obesity and are less responsive to 5-HT_2C_R agonism. **a**–**c**, Body weight curves (**a**), body composition (**b**) and weekly chow intake (**c**) of male WT (*n* = 10) and *Htr2c*^F327L/Y^ mice (*n* = 10) fed on regular chow. **d**–**f**, Cumulative chow intake (**d**), temporal levels of *xy* axis activity (**e**) and *z* axis activity (**f**) during a 2-d period measured by the TSE PhenoMaster apparatus in male WT (*n* = 7) and *Htr2c*^F327L/Y^ (*n* = 9) mice (left). Averaged values during the dark or light cycle on left (right). **g**–**i**, Body weight curves (**g**), body composition (**h**) and weekly HFD intake (**i**) of male WT (*n* = 7) and *Htr2c*^F327L/Y^ (*n* = 9) mice fed on HFD. **j**, Cumulative HFD intake during a 2-d period in male WT (*n* = 7) and *Htr2c*^F327L/Y^ (*n* = 9) mice (left). Averaged HFD intake during the dark cycle, light cycle or 24 h (right). **k**, One-hour food intake in chow-fed male WT (*n* = 8) and *Htr2c*^*F327L/Y*^ (*n* = 8) mice (6 months of age) after i.p. injections of saline or lorcaserin (3 mg kg^–1^). **l**, One-hour food intake in chow-fed male WT (*n* = 9) and *Htr2c*^F327L/Y^ mice (*n* = 14) (2 months of age) after i.p. injections of saline or leptin (5 mg kg^−1^). **m**, Representative traces in POMC neurons from WT and *Htr2c*^F327L/Y^ mice treated with lorcaserin. **n**, Percentage/number of POMC neurons from WT and *Htr2c*^F327L/Y^ mice that were depolarized by or irresponsive to lorcaserin. **o**,**p**, Resting membrane potential, depolarization (**o**) and increases in firing frequency (**p**) induced by lorcaserin in WT (*n* = 18) and *Htr2c*^F327L/Y^ (*n* = 7) mice. Two-way analysis of variance followed by Sidak’s multiple comparisons were performed in **a**,**c**–**f** (left), **g**,**i**–**I**. *P* values in some individual data points in **a**,**c**,**d**–**f** (left), **i**,**j** were determined by two-tailed unpaired Student’s *t*-test. Two-tailed unpaired Student’s *t*-tests were performed in **b**,**d**,**e**,**h**,**j** (right), **f** (left), **o**,**p**. *P < 0.05, **P < 0.01 and ***P < 0.001. Data are presented as mean ± s.e.m. Source data

**Extended Data Fig. 3:**
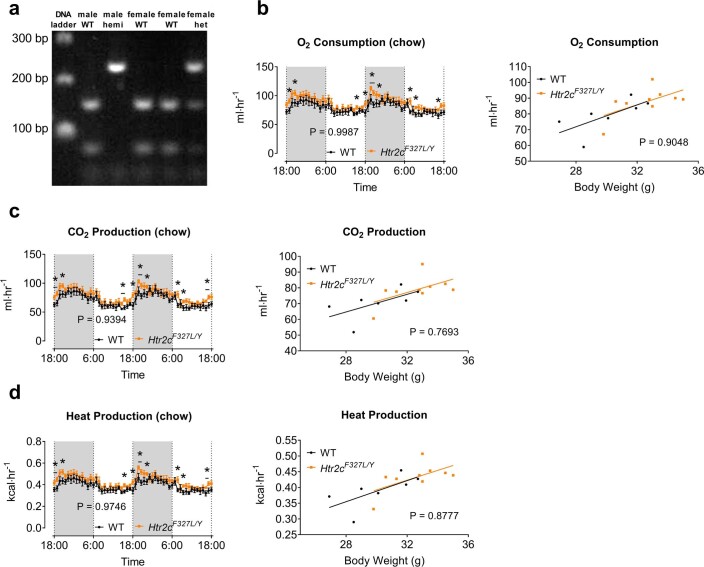
Metabolic phenotype in chow-fed male *Htr2c*^*F327L/Y*^ mice. **a**, The PCR products around the mouse F328 (equivalent to human F327) were amplified from genomic DNA extracts and incubated with NlaIV. A WT allele was cut into 153, 79 and 34 bp bands. A F327L mutant allele resulted in 232 and 34 bp bands. Heterozygous alleles (a WT allele + a F327L mutant allele) resulted in 232, 153, 79 and 34 bp bands (repeated 3 times independently for all groups). **b**-**d**, Left panels: temporal levels of O_2_ consumption (**b**), CO_2_ production (**c**) and heat production (**d**) during a 2-day period measured by the TSE PhenoMaster in 18-week old chow-fed male WT (N = 7) and *Htr2c*^*F327L/Y*^ (N = 9) mice. Right panels: the regression of O_2_ consumption, CO_2_ production and heat production with body mass. Two way ANOVA followed by Sidak’s multiple comparisons were performed in **b** (left panel), **c** (left panel), **d** (left panel). P values in **b** (left panel), **c** (left panel), **d** (left panel) were determined by two-tailed unpaired t-test. Regression-based ANCOVA analysis was performed in **b** (right panel), **c** (right panel) and **d** (right panel). *, p < 0.05. Data are presented as mean ± SEM.*, p < 0.05. Source data

**Extended Data Fig. 4:**
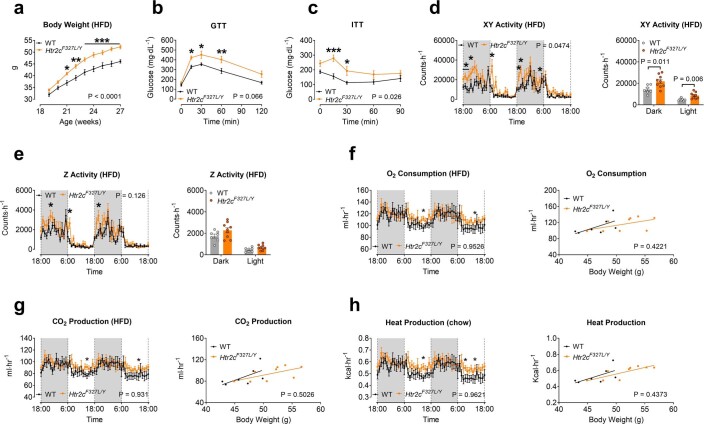
Metabolic phenotype in HFD-fed male *Htr2c*^*F327L/Y*^ mice. **a**, Body weight curves of male WT (N = 7) and *Htr2c*^*F327L/Y*^ mice (N = 9) fed on HFD. **b**-**c**, Blood glucose levels in a glucose tolerance test (GTT) (**b**) or in an insulin tolerance test (ITT) (**c**) in male WT (N = 7) and *Htr2c*^*F327L/Y*^ (N = 9) mice fed on HFD. N = 7 or 9 mice per group. **d**-**e**, Left panels: temporal levels of XY axis activity (**d**), Z axis activity measured by the TSE PhenoMaster in 27-week old HFD-fed male WT (N = 7) and *Htr2c*^F327L/Y^ (N = 9) mice (**e**), Right panels: averaged values during the dark cycle, light cycle or 24 hours. **f**-**h**, Temporal levels of O_2_ consumption (**f**), CO_2_ production (**g**) and heat production (**h**) during a 2-day period measured by the TSE PhenoMaster in 27-week old HFD-fed male WT (N = 7) and *Htr2c*^*F327L/Y*^ (N = 9) mice. Right panels: the regression of O_2_ consumption, CO_2_ production and heat production with body mass. Two way ANOVA followed by Sidak’s multiple comparisons were performed in **a**, **b**, **c**, **d** (left panel), **e** (left panel), **g** (left panel) and **h** (left panel). P values in **d** (left panel), **e** (left panel), **f** (left panel), **g** (left panel) and **h** (left panel) were determined by two-tailed unpaired t-test. Two-tailed unpaired t-tests were performed in **d** (right panel) and **e** (right panel). Regression-based ANCOVA analysis was performed in **f** (right panel), **g** (right panel) and **h** (right panel). *, p < 0.05. Data are presented as mean ± SEM. Source data

To determine whether a human LOF variant affecting 5-HT_2C_R (F327L) can cause obesity, we used a CRISPR-Cas9 approach to generate a knock-in *Htr2c*^F327L^ mouse line (Methods) (Extended Data Fig. 3a). Experiments were performed in heterozygous female mice (carrying a variant on one X chromosome; wild-type gene sequence on the other X chromosome) and hemizygous male mice (carrying a variant on their only X chromosome). When fed with a regular chow diet, male *Htr2c*^F327L/Y^ hemizygous mice showed modest body weight gain compared to wild-type littermates, associated with increased fat mass (Fig. 4a,b). In addition, male *Htr2c*^F327L/Y^ mice exhibited increased food intake compared to wild-type littermates from 7 weeks of age before body weight divergence was apparent (Fig. 4c). We confirmed hyperphagia in male *Htr2c*^F327L/Y^ mice using TSE PhenoMaster metabolic cages (Fig. 4d), which also revealed an increase in physical activity (Fig. 4e,f). No changes were detected in overall O_2_ consumption, CO_2_ and heat production when using body weight as a covariate, whereas subtle increases were detected at intervals during the 2-d recording period in male *Htr2c*^F327L/Y^ mice (Extended Data Fig. 3b–d). When challenged with chronic high fat diet (HFD) feeding, male *Htr2c*^F327L/Y^ mice showed greater body weight gain, associated with increased fat mass and lean mass (Fig. 4g,h and Extended Data Fig. 4a). Consistent with increased adiposity, male *Htr2c*^F327L/Y^ mice displayed impaired glucose tolerance and impaired insulin sensitivity (Extended Data Fig. 4b,c). Male *Htr2c*^F327L/Y^ mice rapidly developed hyperphagia on an HFD before divergence in body weight (Fig. 4i). The TSE PhenoMaster metabolic cage study confirmed increased HFD consumption (Fig. 4j) and revealed increases in physical activity and no changes in O_2_ consumption, CO_2_ and heat production (Extended Data Fig. 4d–h). Collectively, these results indicate that the F327L 5-HT_2C_R variant leads to weight gain in male mice due primarily to hyperphagia, despite increased physical activity.

**Extended Data Fig. 5:**
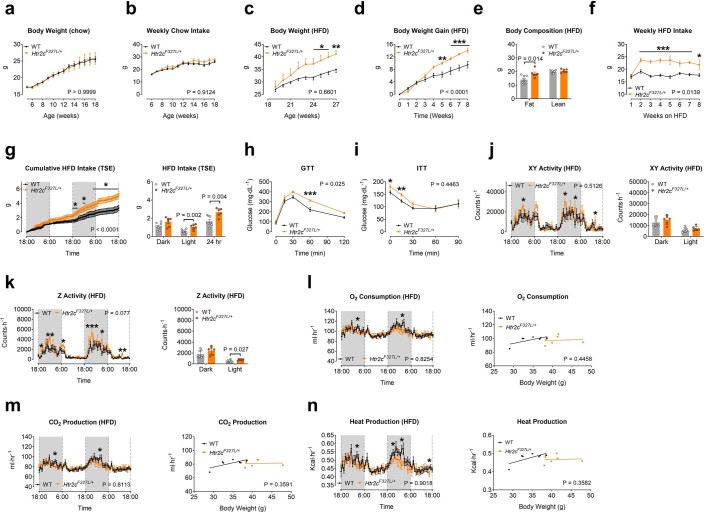
Metabolic phenotype in HFD-fed female *Htr2c*^*F327L/+*^ mice. **a**-**b**, Body weight curves (**a**) and weekly chow intake (**b**) of female WT (N = 8) and *Htr2c*^*F327L/+*^ (N = 8) mice fed on regular chow. **c**-**f**, Body weight curves (**c**), body weight gain (**d**), body composition (**e**) and weekly HFD intake (**f**) of female WT (N = 6) and *Htr2c*^*F327L/+*^ (N = 8) mice fed on HFD. **g**, Left panel: cumulative HFD intake during a 2-day period measured by the TSE PhenoMaster in female WT (N = 6) and *Htr2c*^*F327L/+*^ (N = 8) mice. Right panel: averaged HFD intake during the dark cycle, light cycle or 24 hours. **h**-**i**, Blood glucose levels in a glucose tolerance test (GTT) in female WT (N = 7) and *Htr2c*^*F327L/+*^ (N = 6) mice (**h**) or in an insulin tolerance test (ITT) in female WT (N = 8) and *Htr2c*^*F327L/+*^ (N = 6) mice (**i**). **j**-**n**, Left panels: temporal levels of XY axis activity (**j**), Z axis activity (**k**), O2 consumption (**l**), CO2 production (**m**) and heat production (**n**) during a 2-day period measured by the TSE PhenoMaster in HFD-fed female WT (N = 6) and *Htr2c*^*F327L/+*^ (N = 8) mice. Right panels: averaged values during the dark cycle, light cycle in **j** and **k** and the regression of O_2_ consumption, CO_2_ production and heat production with body mass in **l**, **m** and **n**. Two way ANOVA followed by Sidak’s multiple comparisons were performed in **a**-**d**, **f**, **g**, **h**, **i**, **j** (left panel), **k** (left panel), **l** (left panel), **m** (left panel), **n** (left panel). P values in **c**, **f**, **g** (left panel), **i**, **j** (left panel), **k** (left panel), **l** (left panel), **m** (left panel), **n** (left panel) were determined by two-tailed unpaired t-test. Two-tailed unpaired t-tests were performed in **e**, **g** (right panel), **j** (right panel) and **k** (right panel). Regression-based ANCOVA analysis was performed in **l** (right panel), **m** (right panel) and **n** (right panel). *, p < 0.05, **, p < 0.01, ***, p < 0.001. Data are presented as mean ± SEM. Source data

As the F327L 5-HT_2C_R variant was identified in the heterozygous form in a female proband with obesity, we characterized metabolic phenotypes in female *Htr2c*^F327L/+^ heterozygous mice. When fed on a chow diet, female *Htr2c*^F327L/+^ mice showed comparable body weight and food intake to female wild-type littermates (Extended Data Fig. 5a,b). When fed on HFD, female *Htr2c*^F327L/+^ mice developed hyperphagic obesity associated with glucose intolerance and insulin resistance (Extended Data Fig. 5c–i). Modest increases in physical activity were detected in HFD-fed female *Htr2c*^F327L/+^ mice; there was no change in O_2_ consumption, CO_2_ and heat production (Extended Data Fig. 5j–n). These results indicate that the heterozygous F327L 5-HT_2C_R mutation caused hyperphagic obesity in female mice on a HFD.

### *Htr2c*^F327L^ mice are less responsive to 5-HT_2C_R agonism

**Extended Data Fig. 6:**
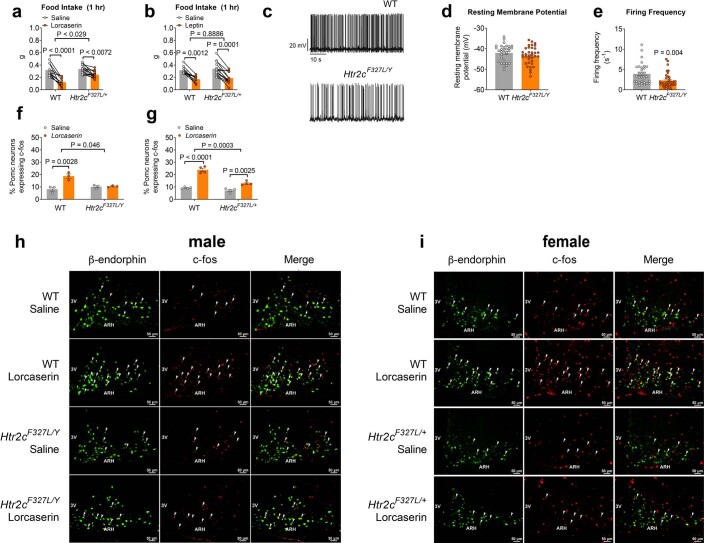
Activity of Pomc neurons in male *Htr2c*^*F327L/Y*^ mice. **a**, One hour food intake in chow-fed female WT (N = 10) and *Htr2c*^*F327L/+*^ mice (2 months of age) (N = 13) after i.p. injections of saline or lorcaserin (3 mg per kg). **b**, One hour food intake in chow-fed female WT (N = 10) and *Htr2c*^*F327L/+*^ mice (2 months of age) (N = 13) after i.p. injections of saline or leptin (5 mg per kg). **c**, Representative current clamp baseline (without any treatment) traces in Pomc neurons from WT and *Htr2c*^*F327L/Y*^ mice. **d**-**e**, Baseline resting membrane potential (**d**) and firing frequency (**e**) in Pomc neurons from WT (N = 31) and *Htr2c*^*F327L/Y*^ (N = 35) mice. **f**-**g**, Percentage of Pomc neurons (labeled by β-endorphin) expressing c-fos in chow-fed male WT (N = 3) and *Htr2c*^*F327L/Y*^ mice (N = 3) (7 months of age) (**f**) and chow-fed female WT (N = 4) and *Htr2c*^*F327L/+*^ mice (N = 4) (4 months of age) (**g**) after i.p. injections of saline or lorcaserin (3 mg per kg). **h**-**i**, Representative microscopic images showing immunoreactivity of β-endorphin (left green), c-fos (middle red) and merge in male (repeated 3 times independently for all groups) (**h**) and female (repeated 4 times independently for all groups) (**i**) mice. Two way ANOVA followed by Sidak’s multiple comparisons were performed in **a**, **b**, **f**, **g**. Two-tailed unpaired t-tests were performed in **d** and **e**. Data are presented as mean ± SEM. Source data

We next examined the effect of a 5-HT_2C_R agonist, lorcaserin, on food intake. Lorcaserin-induced anorexia was blunted in both male *Htr2c*^F327L/Y^ mice and female *Htr2c*^F327L/+^ mice compared to wild-type mice (Fig. 4k and Extended Data Fig. 6a). The F327L mutation did not affect leptin-induced anorexia in either male or female mice (Fig. 4l and Extended Data Fig. 6b). We and others have previously demonstrated that the anorectic effects of 5-HT_2C_R-mediated signaling are largely mediated through POMC neurons^7,23^. Here we found that the F327L 5-HT_2C_R mutation reduced the baseline firing frequency of POMC neurons without altering baseline resting membrane potential (Extended Data Fig. 6c–e). Notably, fewer POMC neurons from *Htr2c*^F327L/Y^ mice were depolarized by lorcaserin (20% in *Htr2c*^F327L/Y^ mice versus 43% in wild-type mice, *P* = 0.0329, chi-squared test; Fig. 4m,n). We found that lorcaserin-induced depolarization and increased firing frequency were attenuated in POMC neurons from mutant mice compared to wild-type mice (Fig. 4o,p). Consistently, lorcaserin-induced c-fos expression in POMC neurons was reduced in both male *Htr2c*^F327L/Y^ mice and female *Htr2c*^F327L/+^ mice (Extended Data Fig. 6f–i).

### *Htr2c*^F327L^ mice exhibit behavioral deficits

**Fig. 5:**
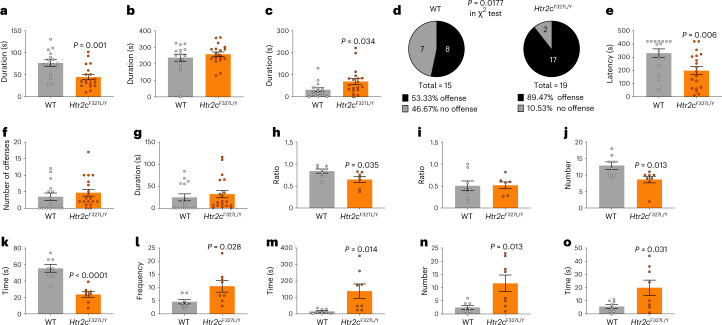
Knock-in male *Htr2c*^*F327L/Y*^ mice develop social anxiety. **a**–**c**, Social exploration time (**a**), non-social exploration time (**b**) and defensive time (**c**) spent by male WT (*n* = 15) and *Htr2c*^F327L/Y^ mice (*n* = 19) (4–5 months of age) in the resident-intruder test. **d**, Percentage/number of male WT and *Htr2c*^F327L/Y^ mice that exhibited offensive behaviors toward the intruder. **e**,**g**, Offensive latency, number and time quantified in male WT (*n* = 15) and *Htr2c*^F327L/Y^ (*n* = 19) mice in the intruder test. **h**, Ratio of time spent sniffing a mouse when interacting with a mouse compared to an object in the three-chamber social interaction test quantified in male WT (*n* = 7) and *Htr2c*^F327L/Y^ (*n* = 8) mice. **i**, Ratio of time spent sniffing a novel mouse when interacting with a new mouse compared to a familiar mouse in the three-chamber social interaction test in male WT (*n* = 7) and *Htr2c*^F327L/Y^ (*n* = 8) mice. **l**,**m**, Number of entries to (**l**) and time spent on (**m**) the open arms of the EPM apparatus by male WT (*n* = 8) and *Htr2c*^F327L/Y^ (*n* = 8) mice. **n**,**o**, Number of (**n**) and time (**o**) spent in head dipping on the open arms of the EPM apparatus by male WT (*n* = 8) and *Htr2c*^F327L/Y^ (*n* = 8) mice. *P* values were determined by two-tailed unpaired Student’s *t*-tests. Data are presented as mean ± s.e.m. with individual data points. Source data

**Extended Data Fig. 7:**
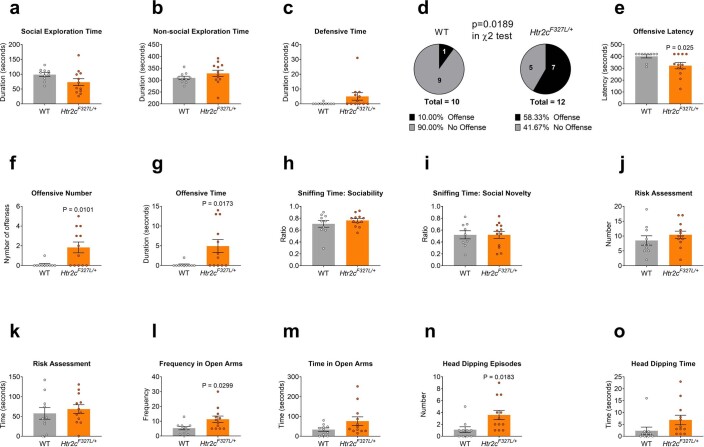
Abnormal behaviour in knock-in female *Htr2c*^*F327L/+*^ mice. **a**-**c**, Social exploration time (**a**), non-social exploration time (**b**) and defensive time (**c**), spent by female WT (N = 10) and *Htr2c*^*F327L/+*^ (N = 12) mice (4 months of age) in the resident-intruder test. **d**, Percentage/number of female WT and *Htr2c*^*F327L/+*^ mice that exhibited offensive behaviors towards the intruder. **e**-**g**, Offensive latency, number and time quantified in female WT (N = 10) and *Htr2c*^*F327L/+*^ (N = 12) mice in the intruder test. **h**, Ratio of time spent sniffing a mouse when interacting with a mouse and an object in the three-chamber social interaction test. N = 9 mice in WT group and 12 mice *Htr2c*^*F327L/+*^ group. **i**, Ratio of time spent in sniffing a novel mouse when interacting with a new mouse and a familiar mouse in the three-chamber social interaction test, N = 9 mice in WT group and 12 mice *Htr2c*^*F327L/+*^ group. **j**-**k**, Number (**j**) and time (**k**) of risk assessment behaviour by female WT (N = 10) and *Htr2c*^*F327L/+*^ mice (N = 12) (4 months of age) in the EPM test. **l**-**m**, Number of entries to (**l**) and time spent on (**m**) the open arms of the EPM apparatus by female WT (N = 10) and *Htr2c*^*F327L/+*^ (N = 12) mice. **n**-**o**, Number of (**n**) and time (**o**) spent in head dipping on the open arms of the EPM apparatus by female WT (N = 10) and *Htr2c*^*F327L/+*^ (N = 12) mice. Two-tailed unpaired t-tests. Data are presented as mean ± SEM. Source data

Serotonin signaling is involved in coordinating defensive responses to threats in experimental animals^24^. We therefore examined the behavior of male *Htr2c*^F327L/Y^ mice in a resident-intruder test. In this test, a male ‘intruder’ mouse is placed in the home cage of a ‘resident’ mouse (male wild-type or *Htr2c*^F327L/Y^) that has been individually housed. *Htr2c*^F327L/Y^ mice showed reduced social exploration, whereas non-social exploration was not altered (Fig. 5a,b and Methods). *Htr2c*^F327L/Y^ mice showed significantly increased defensive behaviors compared to wild-type littermates (Fig. 5c) and increased offensive attempts toward the intruder (89.5% in *Htr2c*^F327L/Y^ mice versus 53.3% in wild-type mice, *P* = 0.0177, chi-squared test) with reduced latency (Fig. 5d,e) although the average number of offensive episodes and time spent in these behaviors was comparable between male *Htr2c*^F327L/Y^ and wild-type mice (Fig. 5f,g). In a three-chamber social interaction test, compared to male wild-type mice, male *Htr2c*^F327L/Y^ mice displayed reduced interaction with another mouse (Fig. 5h), demonstrating reduced sociability. Notably, both groups showed comparable preference to interact with a novel mouse versus a familiar mouse (Fig. 5i), excluding neophobia as a potential confounder in this test. The elevated plus-maze (EPM) test is classically used to assess anxiety-related behavior and screen anxiolytic compounds in rodents^25^. In this test, male *Htr2c*^F327L/Y^ mice paradoxically showed reduced risk assessment behavior before they entered the open arms (Fig. 5j,k) and spent more time in the open arms (Fig. 5l,m), whereas on the open arms, *Htr2c*^F327L/Y^ mice showed significantly increased head dipping (Fig. 5n,o), an exploratory behavior that can be induced by antagonists of 5-HT_2C_R^26^. Female *Htr2c*^F327L/+^ mice partially recapitulated the behaviors seen in male *Htr2c*^F327L/Y^ mice, including increased offensive behavior in the resident-intruder test, increased entry into the open arms and increased number of head dipping episodes on the EPM apparatus, whereas social behavior and risk assessment behavior were not altered (Extended Data Fig. 7). Previous studies have shown that *Htr2c* knockout mice exhibit impaired performance in the Morris water maze test of spatial learning, with reduced aversion to a novel environment^27^. Accordingly, we consider that the spectrum of behavioral changes seen in the mouse model of a human *HTR2C* mutation (reactive aggression, locomotor hyperactivity and anxiety) are modulated by the coexistence of cognitive deficits that impair the animals’ ability to learn how to respond to environmental challenges.

## Discussion

We provide evidence for a functional role of rare variants in *HTR2C* (encoding 5-HT_2C_R) in the development of obesity in humans. These findings have both diagnostic and therapeutic implications. In contrast to some monogenic obesity syndromes, which display classical Mendelian inheritance (*LEP*, *LEPR*, *POMC* and *PCSK1*), rare heterozygous variants in *HTR2C* are generally not fully penetrant. Our results align with findings reported for an increasing number of genes (*KSR2* (ref. ^28^), *MRAP2* (ref. ^29^) and *SRC-1* (ref. ^30^)) where complete disruption in mice causes obesity but where the penetrance of heterozygous rare LOF variants in humans is more variable and likely to be modified by other genetic and environmental factors.

There are a number of limitations associated with our human studies. Given the rarity of the variants identified here and the fact they do not display Mendelian inheritance, association studies in tens of thousands of individuals with severe obesity versus a comparable number of control individuals will be needed to find statistical evidence for enrichment^31,32^; however, to our knowledge, no clinical cohort of this size currently exists. Association studies in very large population-based cohorts such as the UK Biobank can be informative, but people carrying pathogenic variants in the gene encoding 5-HT_2C_R are likely to be under-represented in such cohorts^33^, as exemplified by studies of other genes involved in severe obesity, for example *MC4R*^34,35^. Further analysis is warranted in larger cohorts, but additional experimental evidence (for example using animal models of human disease) is often needed to establish the causal role of rare variants with variable penetrance. Animal studies also have limitations. For example, the laboratory setting where metabolic and behavioral phenotypes were characterized does not fully model the complex nutritional and social environment people live in.

We observed that anxiety and maladaptive behavior are frequently reported in a large cohort of children with severe obesity, findings that suggest shared mechanistic origins for these disorders; however, as a medical history of these conditions is sometimes not obtained, or if obtained it is attributed to weight stigma or family circumstances, their true prevalence may be underestimated in this study. Further studies of large clinical cohorts in whom the prevalence of neurobehavioral conditions is measured using standardized clinical questionnaires are needed. Our findings indicate that a careful history of anxiety, mood disturbances and maladaptive behavior should be obtained in people presenting with severe early-onset obesity as positive clinical findings may guide genetic testing and have relevance for treatment.

In keeping with studies in mice which showed that the anorectic effects of 5-HT are mediated by 5-HT_2C_Rs expressed on hypothalamic POMC neurons^6,7^, we show that knock-in mice carrying F327L 5-HT_2C_R have impaired depolarization of POMC neurons when stimulated by the serotoninergic agonist, lorcaserin. As such, severe obesity in some people with functional variants in *HTR2C* is likely to be driven by impaired melanocortin signaling. These findings have clinical relevance as a melanocortin 4 receptor agonist, setmelanotide, causes weight loss in people with impaired melanocortin signaling due to *POMC*, *PCSK1* and *LEPR* mutations^9–11^ and is being trialed (ClinicalTrials.gov identifier NCT04963231) in people with rare variants in other genes that regulate the depolarization of POMC neurons and the transcription of *POMC* (Fig. 2). Clinically meaningful weight loss in people carrying *HTR2C* variants receiving an MC4R agonist may provide biological validation of the pathogenicity of specific variants and inform the inclusion of *HTR2C* in diagnostic gene panels for severe childhood-onset obesity^36^.

In our studies, knock-in mutant mice displayed increased food intake and locomotor hyperactivity as seen in knockout mice;^37,38^ there was minimal difference in energy expenditure. The metabolic and behavioral phenotype of knock-in mice was more marked in hemizygous males (minimal functioning 5-HT_2C_Rs due to the presence of the mutation on their single X chromosome) compared to heterozygous female knock-in mice (one wild-type X chromosome and one mutant X chromosome). Modest differences in phenotype are likely to be explained by a gene-dosage effect, although other explanations for sex-specific differences cannot be excluded. It is noteworthy that 16 of 19 probands in whom *HTR2C* variants were identified in our study were female, whereas 50% of the GOOS cohort who were screened were female. Mutations in genes on the X chromosome have different consequences in males and females. To date, more than 100 X-linked inherited diseases have been identified; the majority are recessively inherited, a much smaller number are dominantly inherited and a few are dominant and lethal in hemizygous males. The excess of affected female heterozygotes compared to male heterozygotes in this study is suggestive of X-linked dominant inheritance. X-linked dominant disorders that are lethal in males in utero are, by definition, seen only in female heterozygotes, with affected (hemizygous) males manifesting as an excess of spontaneous miscarriages in affected families as seen in Rett syndrome. In view of the more severe metabolic and behavioral phenotype seen in male versus female mice, the low prevalence of males carrying *HTR2C* variants seen in this study might be explained by excess mortality in affected males. Further studies to establish the frequency of miscarriage in these families will be needed to explore this further.

5-HT_2C_Rs are expressed along ascending dopaminergic pathways and their activation in regions such as the nucleus accumbens regulates inhibitory control over dopamine release^39,40^ suggesting a plausible mechanism underlying hyperactivity^41^. In a series of behavioral studies, we found that male hemizygous *Htr2c*^F327L/Y^ and female heterozygous *Htr2c*^F327L/+^ mice exhibited decreased social exploration and increased aggression toward an intruder. 5-HT plays an evolutionarily conserved role in the regulation of social and emotional behaviors in many species;^24,39,42–44^ mice given selective serotonin reuptake inhibitors that enhance central 5-HT levels, show reduced aggression^45^. Neural circuits in the amygdala (a prominent site of 5-HT_2C_R expression) play a critical role in linking fear processing and defensive responses (freeze/flight)^43,46^, which enable a rapid response to a natural predator, promoting survival.

Clinically, anxiety disorders (including social anxiety) are generally considered an extreme manifestation of the anxiety response to perceived threats. Several epidemiological studies have found that anxiety is a common feature in people living with obesity and particularly in women with obesity^47^. To date, the assumption has been that this association may reflect the widespread stigma and discrimination people with obesity face and/or early life experiences which influence susceptibility to obesity and anxiety; however, our findings suggest another possible explanation. In some people (particularly women), impaired serotonin signaling may directly contribute to both their obesity and their social anxiety/maladaptive behavior. Other serotonin receptors may also contribute to human neuropsychiatric behaviors. Goldman and colleagues^48^ identified an LOF mutation in 5-HT_2B_R in an isolated Finnish extended family with a strong history of aggressive behavior and impulsivity. In line with these clinical observations, they showed that mice lacking 5-HT_2B_R exhibited impulsivity and aggressive behavior in a resident-intruder paradigm. While LOF variants in the gene encoding 5-HT_2C_R are rare, other genes and/or mechanisms which perturb signaling through 5-HT_2C_R may contribute to obesity and anxiety in some people. Indeed, selective serotonin reuptake inhibitors are effective in reducing symptoms of anxiety and specifically are first-line treatment for panic disorder, obsessive-compulsive disorder and social anxiety disorder. Our findings suggest that enhanced signaling through 5-HT_2C_R could explain the clinical effectiveness of these drugs in the treatment of a range of disorders characterized by anxiety. Further studies are needed to identify additional regulators of 5-HT_2C_R expression and signaling, which may further add to our understanding of the association between obesity and anxiety. Targeting the mechanisms that regulate both canonical and β-arrestin-mediated signaling by 5-HT_2C_R could inform the development of medications that are both effective and safe for chronic weight management.

## Methods

### Human studies

These studies were approved by the Multi-regional Ethics Committee and Cambridge Local Research Ethics Committee (03/103, 03/104 and 18/EE/0032) and conducted in accordance with the principles of the Declaration of Helsinki. Each participant or their legal guardian (for children under 16 years of age) provided written, informed consent and minors provided verbal or written consent.

We studied people with severe obesity (BMI (kg m^−2^) SDS > 3) of early-onset (<10 years of age) recruited to the GOOS cohort (www.goos.org.uk) between 1997 and 2022. Referring physicians completed standardized questionnaires that captured medical history, family history and findings on clinical examination, including anthropometric data and the results of investigations. The sex of participants was assigned by clinical examination by physicians. To describe the prevalence of neurobehavioral conditions in the GOOS cohort, we excluded people in whom genetic obesity syndromes had been identified. As a result data on 7,775 people were included. Exome sequencing and targeted resequencing were performed in a subset of the cohort as previously described;^12,13^ all variants referred to in this study were verified by Sanger sequencing.

Probands and their families were invited to participate in physiological studies at the Wellcome-MRC Institute of Metabolic Science Translational Research Facility, Addenbrooke’s Hospital. Weight and height were measured barefoot in light clothing. Dual X-ray absorptiometry (DPX software; Lunar Corp) was used to determine body composition. BMR was determined by indirect calorimetry after a 10-h overnight fast using an open circuit, ventilated, canopy measurement system (Europa Gas Exchange Monitor; NutrEn Technology). BMR adjusted for body composition was compared to predicted metabolic rate based on standard age and sex-specific equations. Blood pressure was measured in the rested fasted state using wrist monitors (OMRON Healthcare). For the analysis of insulin and glucose, patients with type 2 diabetes were excluded. Blood samples were taken in the fasted state for all assays.

### UK Biobank 200K OQFE exomes and clinical phenotypes

This research was conducted using the UK Biobank Resource^22^ under application no. 53821. We used pVCF variant file (chrX, block 17) from OQFE exome pipeline (UK Biobank Field 23156; *n* = 200,629 exomes available to us). Reported sex was obtained from Field 31 (Supplementary Table 2). We split and left-normalized multiallelic entries (bcftools v.1.9) and defined variant consequences with respect to Ensembl canonical transcript ENST00000276198 using Ensembl Variant Effect Predictor (Ensembl release v.102).

In females, we used genotype calls provided in the pVCF file. In males, we treated any non-homozygous-REF genotype as hemizygous, consistent with reported allelic depths. Relatedness was obtained from the UK Biobank (ukbgene rel) and one person was excluded from each related pair among all the OQFE exomes (kinship ≥ 0.0442, KING, third-degree kinship or closer; retained pairs contained within OQFE exomes and excluded individuals in column ‘ID2’; *n* = 15,547 people). Exomes were restricted European genetic ethnic grouping (Field 22006, self-reported ‘White British’ and tight cluster in genotype principal-component analysis). Unrelated European OQFE exomes were taken forward for analysis (*n* = 153,352).

We obtained BMI (kg m^−2^) from the UK Biobank initial assessment visit (Field 21001, Instance 0), available to us for *n* = 152,837 of 153,352 unrelated European exomes (83,864 females and 69,488 males). To investigate phenotypes related to anxiety or mood, we used the mental distress (‘Mental health problems ever diagnosed by a professional’, Field 20544) section of online follow-up Mental Health questionnaire (category 136) (rationale and design is provided at https://biobank.ctsu.ox.ac.uk/showcase/refer.cgi?id=22), which had an available ‘Date of completing’ (Field 20400) for *n* = 53,174 of 153,352 unrelated European exomes. Mental distress data provide self-reported professional diagnoses in 16 categories (https://biobank.ctsu.ox.ac.uk/showcase/field.cgi?id=20544); limitations include lack of complete data (returned questionnaires), ascertainment bias and self-reporting of professional diagnoses. Of 16 categories of mental distress, we selected three categories of interest: ‘psychological over-eating or binge-eating’, ‘anxiety, nerves or generalized anxiety disorder’ (anxiety) and ‘depression’, and for selected variants we further inspected the reporting of any category of mental distress in people who carried variants and those who did not.

### Single-variant association analysis with BMI, obesity and severe obesity

Association analysis of single variants with allele count >20 was performed separately for females and males among unrelated European exomes (plink v.2.00a1LM; www.cog-genomics.org/plink/2.0/). Case–control association with severe obesity (BMI > 40 kg m^−^^2^) or obesity (BMI > 30 kg m^−2^) was performed using plink2 –glm with Firth regression; *n* = 2,725 individuals (1,868 females and 857 males) were severely obese; *n* = 36,304 individuals (19,018 females and 17,286 males) were obese. Association with BMI as a continuous trait was performed using plink2 –glm. Covariates were age (Field 21003) and 40 genetic principal components (Fields 22009.0.1–40). At very rare variants (allele count <20), ORs were calculated for the number of variant carriers using Fisher’s exact test.

### Rare variant association tests

We performed gene-based association tests separately for males and females using non-synonymous exonic or splicing variants and severe obesity (BMI > 40 kg m^−2^), BMI > 30 kg m^−2^ and for continuous BMI. Gene-based burden and SKATO tests were performed using the SKATBinary_Robust function for dichotomized BMI or the SKAT function for continuous BMI, in R package SKAT v.2.0.1 (method = ‘Burden’ or ‘SKATO’ with default settings). Null models were calculated using SKAT_Null_Model(y~X) where covariates matrix (*X*) contained age (Field 21003.0.0), sex (Field 31.0.0), ten genetic principal components (Fields 22009.0.1–10) and sequencing batch (UKB 50K or 150K exomes). Single-variant *P* values reported from gene-based analyses are SKATBinary_Robust output values for p.value_singlevariant. ORs were also calculated for the number of variant carriers using Fisher’s exact test.

### cDNA constructs and site-directed mutagenesis

The human WT HTR2C complementary DNA (Ensembl transcript ENST00000371951; unedited, INI isoform) was cloned into the pcDNA3.1(+) vector (Invitrogen) and a sequence encoding a FLAG tag was inserted downstream of the sequence encoding the receptor’s signal peptide using a Q5 site-directed mutagenesis kit (New England Biolabs). The edited VSV isoform was generated by changing adenosines at positions A, B, C and D to guanosines^20^, using the QuickChange II XL site-directed mutagenesis kit (Agilent). For β-arrestin protein–protein interaction assays, the receptor VSV isoform lacking a FLAG tag was cloned into the pBiT1.1-C (TK/LgBiT) vector and cDNAs encoding human β-arrestin-1 and -2 were ligated in pBiT2.1-N (TK/SmBiT) vectors (Promega) as described before^49^. Variant constructs of both backbones were generated using QuickChange II XL site-directed mutagenesis kit (Agilent). All constructs were verified using Sanger sequencing.

### Cell culture and transfection

HEK293 or HEK293SL cells were maintained in high glucose Dulbecco’s modified eagle medium (Gibco, 31966), supplemented with 10% fetal bovine serum (Gibco, 10270, South America origin), 1% GlutaMAX (100×) (Gibco, 35050) and 100 U ml^−1^ penicillin and 100 μg ml^−1^ streptomycin (Sigma-Aldrich, P0781) and cultured at 37 °C in humidified air containing 5% CO_2_. Cells were transfected using Lipofectamine 2000 (Gibco, 11668) in serum-free Opti-MEM (Gibco, 31985) according to the manufacturer’s protocol.

### Inositol triphosphate turnover assay

HEK293 cells were seeded in 96-well plates at a density of 40,000 cells per well and transiently transfected on the next day with 5 ng per well WT HTR2C or variant cDNA construct. After transfection, cells were cultured overnight in growth medium supplemented with 5 μl per well [^3^H]-myo-inositol (PerkinElmer, NET115600). Cells were washed once with Hank’s balanced salt solution (HBSS; Gibco, 14025) and subsequently stimulated with 20× 5-hydroxytryptamine stock solution (5-HT; Sigma-Aldrich, 85036) in HBSS containing 10 mM LiCl (Sigma-Aldrich, L9650) for 90 min at 37 °C. Cells were stimulated with different concentrations of 5-HT, ranging from 0 to 10^−5 ^M. After aspirating the stimulation buffer, cells were lysed on ice for 30 min using 50 μl per well 10 mM formic acid (Sigma-Aldrich, F0507). Then 20 μl of lysate was transferred to a white 96-well plate containing 80 μl per well 12.5 mg ml^−1^ yttrium silicate poly-lysine-coated scintillation proximity beads (PerkinElmer, RPNQ0010) in ddH_2_O. Plates were sealed, shaken vigorously for 5 min and the relative amount of radiolabeled inositol monophosphate (IP_1_) was quantified after 8 h of settle time using a TopCount 9012 Microplate Counter (Packard). Results were analyzed using GraphPad Prism 8 (GraphPad Software). Sigmoidal dose–response curves with variable slope (four-parameter logistic regression) were plotted. Results from each assay were normalized to mean counts from the bottom of the 5-HT_2C_R WT curve set as basal and the top value of the 5-HT_2C_R WT curve (maximal efficacy; E_max_) as 100%. Cells transfected with pcDNA3.1(+) (mock) were used as a negative control. Normalized data for each experiment were merged and presented as sum curves, results are from 3–4 independent experiments.

### β-arrestin protein–protein interaction assay

Coupling between 5-HT_2C_R and β-arrestin-1 or β-arrestin-2 was quantified using the NanoBiT protein–protein interaction assay (Promega, M2014). Assays were performed in HEK293SL cells seeded in poly-d-lysine coated, white 96-well plates (15,000 cells per well) and transiently transfected with 50 ng per well of each of the two constructs as described above. As a negative control, the β-arrestin-SmBiT constructs were substituted with the HaloTag-SmBiT negative control vector. The day after transfection and half an hour before assay, culture medium was substituted for 70 μl per well serum-free Opti-MEM (Gibco, 31985). Nanoluciferase activity was measured at 37 °C and in the presence of 5% CO_2_ using a Spark 10 M microplate reader (Tecan). After measurement of the background signal, 20 μl per well Nano-Glo Live Cell Assay System (Promega, N2013) was added and basal luciferase activity was measured for 10 min in 30-s intervals. Subsequently, cells were stimulated with 10 μl of 10 × 5-HT stock solution and the luminescent signal was quantified for 20 min in 30-s intervals. Dose–response curves (sigmoidal with variable slope) were plotted from total peak area under the curve values calculated from each 5-HT concentration, ranging from 0 to 10^−^^5 ^M. Normalized data from independent experiments were merged and presented as sum curves. Results are from 4–5 independent experiments.

### Cell surface expression ELISA

HEK293 cells seeded onto poly-d-lysine coated 96-well plates (40,000 cells per well) were transfected with 5 ng per well of FLAG-tagged *HTR2C* constructs as described above. The day after transfection, the cells were fixed with 3.7% paraformaldehyde in PBS for 15 min at room temperature and washed three times with PBS. Subsequently, nonspecific binding sites were blocked with 3% non-fat dry milk in 50 mM Tris-PBS pH 7.4 (blocking buffer) for 1 h at room temperature. Cells were incubated with a mouse monoclonal anti-FLAG antibody (Sigma-Aldrich, F1804), diluted 1,000× in blocking buffer overnight at 4 °C. Next, cells were washed three times with PBS and incubated with goat anti-mouse IgG (H + L)-HRP conjugate (Bio-Rad Laboratories, 172-1011) (1:1,250 dilution in 1.5% non-fat dry milk in 50 mM Tris-PBS) for 2 h at room temperature. Finally, cells were washed three times with PBS and the chromogenic substrate 3,3′,5,5′-tetramethylbenzidine (TMB CORE+, Bio-Rad Laboratories, BUF062) was used to detect HRP activity. The reaction was stopped with 0.5 M H_2_SO_4_ and absorbance at 450 nm was quantified using a Tecan Infinite M1000 PRO microplate reader.

### Radioligand binding assay

HEK293 cells were seeded in solid white 96-well plates coated with poly-d-lysine and transiently transfected with 50 ng per well FLAG-tagged *HTR2C* constructs. The day after transfection, cells were washed once and incubated on ice with binding buffer (50 μl per well; 20 mM HEPES pH 7.4, 118 mM NaCl, 4.7 mM KCl, 5 mM MgCl_2_, 5.5 mM d-glucose and 0.1% BSA) for 30 min. Next, varying doses of unlabeled 5-HT (0 M to 10^−4 ^M) were added to the cells, immediately followed by 50 μl per well of the ^3^H-labeled 5-HT_2C_R antagonist mesulergine (PerkinElmer, NET1148; 1:5,000 dilution in binding buffer) and cells were incubated on ice for 3 h. After washing twice with ice-cold binding buffer, 20 μl per well 0.1 M NaOH was dispensed and plates were shaken, followed by adding 80 μl per well MicroScint-20 scintillation fluid (PerkinElmer, 6013621). Plates were shaken and activity of bound ^3^H-mesulergine was quantified after a 3 h settle time, using a TopCount 9012 Microplate Counter (Packard).

### Immunofluorescence

HEK293SL cells (10,000 cells per well) were seeded onto poly-d-lysine coated CellCarrier-96 Ultra plates (PerkinElmer, 6055302) and transfected on the following day with 40 ng per well FLAG-tagged constructs as described above. The next day, the cells were fixed with 3.7 % paraformaldehyde in PBS for 15 min and washed twice with PBS. Nonspecific antibody binding sites were blocked with blocking buffer (3 % BSA in PBS) for 1 h. The cells were then incubated with primary antibody (mouse monoclonal anti-FLAG; Sigma-Aldrich, F1804; 1:100 dilution in blocking buffer) for 1 h. After three washing steps with PBS, cells were incubated with secondary antibody (Alexa Fluor 488 goat anti-mouse IgG (H + L); Life Technologies A11029; 1:400 in blocking buffer) for 1 h in the dark. Plates were washed thrice with PBS and cells were permeabilized with 0.1 % Triton X-100 (VWR, 306324 N) in PBS for 5 min. In parallel experiments to capture intracellular receptors, cells were permeabilized before adding the primary anti-FLAG antibody together with an anti-calreticulin antibody, to visualize the ER (Invitrogen, PA3-900, 1:100 dilution), which was recognized using a Alexa Fluor 647 donkey anti-rabbit IgG (H + L) (Thermo Fisher Scientific, A-31573) at a 1:400 dilution in blocking buffer. Next, cell nuclei were stained with DAPI (Merck, D9542; 0.1 μg ml^−1^ final concentration in PBS) for 10 min, followed by three washing steps with PBS. Finally, cells were incubated with DyLight 554 Phalloidin (Cell Signaling Technology, 13054; 1:200 dilution in PBS) for 15 min to stain cytoskeletal F-actin. Cells were washed twice with PBS and stored in the dark at 4 °C before imaging on an Opera Phenix High-Content Screening System (PerkinElmer). For high-content analysis of WT and mutant protein levels at the cell surface and in the cytoplasm, the PerkinElmer Harmony software was used (HH17000012, version 5.0); the DyLight Phalloidin and DAPI channels were used to identify cells and the signal strength of the 488 nm channel was calculated per cell. For ER localization, the overlap between the calreticulin and the 488 nm channel was quantified. Each condition was performed in four technical replicates (on average 400 cells per well) and the mean intensity of all cells per well was calculated. Results shown are from three independent experiments.

### Approval for studies in mice

Care of animals and procedures were approved by the Baylor College of Medicine Institutional Animal Care and Use Committee.

### Generation of *Htr2c*^F327L^ mice

The F327 amino acid residue in the human 5-HT_2C_R protein is equivalent to the F328 of the mouse 5-HT_2C_R protein. We generated a knock-in mouse with the F328L mutation but referred it as *Htr2c*^F327L^ for simplicity. A single-guide RNA (sgRNA) sequence (5′-TTTCATCACCAATATCCTGT) was selected to target the mouse Htr2c gene encoding the F328 and the sgRNA was purchased from Synthego. Cas9 Nuclease was purchased from IDT (Alt-R S.p. Cas9 Nuclease V3). The donor ssDNA template to introduce the F328L point mutation and a silent mutation C326C to remove the restriction site for NlaIV were purchased from IDT. The sequence of ssDNA is as follows: 5′-AATGAGAAGAAAGCTTCCAAAGTCCTTGGCATTGTATTCTTTGTGTTTCTGA TCATGTGGTGTCCGCTTTTCATCACCAATATCCTGTCGGTGCTTTGTGGGAAGGCCTGTAACCAAAAGCTAATGGAGAAACTTCTCAATGTGTTTGTTTGGATT. The BCM Genetically Engineered Murine Model Core microinjected Cas9 (20 ng μl^−1^), ssDNA (20 ng µl^−1^) and sgRNA (20 ng μl^−1^) into the pronuclei of 139 one-cell stage C57BL/6J embryos. Founder animals (F_0_) were identified by PCR-based restriction digestion to detect the CRISPR generated point mutations in the Htr2c gene. PCR product was amplified with the primer pairs: 5′-ACGTCGAAAGAAGAAAGAAAAGC and 5′-GGTAAATTTTGTTGAAGAGAGTGTAC. The 266-bp PCR products were then digested with NlaIV. After the digestion, 153, 79 and 34-bp fragments could be detected for PCR products from a WT allele; 232 and 34 bp fragments could be detected from a mutant *Htr2c*^F327L^ allele. Three independent lines were sequenced for the further confirmation of the point mutation. One of these lines was crossed to C57BL/6J to produce study cohorts. In some breeding, the POMC-CreER/Rosa26-LSL-td TOMATO alleles were introduced to allow specific labeling of POMC neurons.

### Food intake, body weight and body composition

Male and female WT and *Htr2c*^F327L^ littermates were singly housed from 5 weeks of age. Mice were fed ad libitum with a regular chow diet (5V5R-Advanced Protocol PicoLab Select Rodent 50 IF/6F, PicoLab) from weaning to 18 weeks of age and then fed with a HFD (60% fat, no. D12492i, Research Diets) from 18 weeks to 27 weeks of age. Body weight and food intake were measured weekly. Fat mass and lean mass were determined by quantitative magnetic resonance at 18 weeks of age in male mice and at 27 weeks of age in both male and female mice.

### Glucose and insulin tolerance tests

For glucose tolerance tests, after an overnight fast, mice received intraperitoneal (i.p.) injections of 1 g kg^−1^ d-glucose (G8270, Sigma) at 10:00. Blood glucose was measured from tail blood using a glucometer (OneTouchUltra) at 0, 15, 30, 60 and 120 min. For insulin tolerance tests, after a 4-h fast to empty the stomach, mice received i.p. injections of insulin (0.75 U kg^−1^). Blood glucose was measured at 0, 15, 30, 60 and 90 min.

### TSE PhenoMaster metabolic cages

Male mice were acclimated into the TSE PhenoMaster system at 18 weeks (the end of chow feeding period) and again at 27 weeks (the end of HFD feeding period). Female mice were acclimated to the TSE PhenoMaster system at 27 weeks (the end of HFD feeding period). After acclimation for 2 d, food intake, O_2_ consumption, CO_2_ production, heat production, *xy* axis and *z* axis movements were continuously monitored for 4 d and data collected from the last 2 d were used for analysis. O_2_ consumption, CO_2_ production and heat production were analyzed using the online CalR tool^50^.

### Lorcaserin-induced anorexia and c-fos expression in POMC neurons

Male WT and *Htr2c*^F327L/Y^ mice (at 6 months old) and female WT and *Htr2c*^F327L/+^ mice (at 2 months old) were briefly fasted for 2 h (16:00 to 18:00). Then, these mice received i.p. injections of saline or lorcaserin (3 mg kg^−1^) at 18:00. Food was provided to the cages immediately after the injections and food intake was measured for 1 h. Three days later, these mice were subjected to the same protocol with lorcaserin or saline injections in a crossover fashion.

Male WT and *Htr2c*^F327L/Y^ mice (at 7 months old) and female WT and *Htr2c*^F327L/+^ mice (at 4 months old) were briefly fasted for 2 h (14:00 to 16:00) to empty the stomach and then received i.p. injections of saline or lorcaserin (3 mg kg^−1^). One hour later, mice were anesthetized with inhaled isoflurane and quickly perfused with saline, followed by 10% formalin. The brain sections were cut at 25 µm and collected into five consecutive series. One series of brain sections were blocked with 3% normal donkey serum for 1 h incubated with rabbit anti-β-endorphin antibody (1:10,000 dilution; no. H-02233, Phoenix Peptide) and mouse anti-c-Fos antibody (1:1,000 dilution, Ab208942, Abcam) on shaker at room temperature for overnight, followed by the donkey anti-rabbit Alexa Fluor 488 (1:200 dilution, A21206, Invitrogen) and donkey anti-mouse Alexa Fluor 594 (1:200 dilution, A21203, Invitrogen) for 2 h. Slides were cover-slipped and analyzed using a fluorescence microscope. The numbers of β-endorphin-positive cells and c-Fos/β-endorphin double positive neurons in the arcuate nucleus of the hypothalamus were counted in all brain sections and the ratio of c-Fos/β-endorphin double positive neurons to β-endorphin-positive neurons was used to reflect the data value for that mouse. Three mice were included in each group for statistical analyses.

### Leptin-induced anorexia

Male WT and *Htr2c*^F327L/Y^ mice (at 6 months old) and female WT and *Htr2c*^F327L/+^ mice (at 2 months old) were briefly fasted for 2 h (16:00 to 18:00). Then, these mice received i.p. injections of saline or leptin (5 mg kg^−1^) at 18:00. Food was provided to the cages immediately after the injections and food intake was measured for 1 h. Three days later, these mice were subjected to the same protocol with leptin or saline injections in a crossover fashion.

### Electrophysiology

Male POMC-CreER/Rosa26-LSL-tdTOMATO and POMC-CreER/Rosa26-LSL-td TOMATO/*Htr2c*^F327L/Y^ littermates at 5–6 months old were used for recordings from POMC neurons in the ARH. Mice were anesthetized with isoflurane and were transcardially perfused with a modified ice-cold sucrose-based cutting solution (pH 7.4; containing 10 mM NaCl, 25 mM NaHCO_3_, 195 mM sucrose, 5 mM glucose, 2.5 mM KCl, 1.25 mM NaH_2_PO_4_, 2 mM sodium pyruvate, 0.5 mM CaCl_2_ and 7 mM MgCl_2_, bubbled continuously with 95% O_2_ and 5% CO_2_). The mice were then decapitated and the entire brain was removed and immediately submerged in the cutting solution. Coronal slices (220 μm) were cut with a Microm HM 650V vibratome (Thermo Scientific). Brain slices containing the ARH were collected and recordings were made at levels throughout this brain region. The slices were recovered for ~ 30 min at 32 °C and then maintained at room temperature for another 1 h in oxygenated (95% O_2_ and 5% CO_2_) artificial cerebrospinal fluid (pH 7.4; containing 126 mM NaCl, 2.5 mM KCl, 2.4 mM CaCl_2_, 1.2 mM NaH_2_PO_4_, 1.2 mM MgCl_2_, 11.1 mM glucose and 21.4 mM NaHCO_3_) before recording.

Slices were transferred to the recording chamber at 32 °C and perfused continuously with oxygenated artificial cerebrospinal fluid at a flow rate of 1.8–2.0 ml min^−1^. Slices were allowed to equilibrate for at least 5 min before recording. tdTOMATO-labeled neurons in the ARH were visualized using epifluorescence and infrared–differential interference contrast (IR–DIC) imaging on an upright microscope (Eclipse FN-1, Nikon) equipped with a moveable stage (MP-285, Sutter Instrument). Patch pipettes with resistances of 3–5 MΩω were filled with intracellular solution (pH 7.3) containing 128 mM potassium gluconate, 10 mM KCl, 10 mM HEPES, 0.1 mM EGTA, 2 mM MgCl_2_, 0.05 mM GTP (sodium salt) and 0.05 mM ATP (magnesium salt). Recordings were made using a MultiClamp 700B amplifier (Axon Instruments), filtered at 1 kHz and sampled at 10 kHz using Digidata 1440A and analyzed offline with pClamp v.10.3 software (Axon Instruments). Series resistance was monitored during the recording and the values were generally <10 MΩ and were not compensated. The liquid junction potential was monitored and corrected. Data were excluded if the series resistance exceeding 20% change during the experiment or without overshoot for action potential. Current clamp was engaged to test neural firing frequency and resting membrane potential at the baseline and after puff delivery of lorcaserin (5 s at 30 μM). To ensure that each recorded neuron receive same amount of lorcaserin, the neurons located on the surface of the slice were selected for recording and the puff pipette was always put at a 100 μm horizontal and 100 μm vertical distance from the recorded neurons. The puff strength was maintained at a same level using a repeatable pressure pulse system (Picospritzer III, Parker). Each neuron was recorded at least 1 min baseline and only the neurons with stable baseline were used to test the lorcaserin treatment. The values of resting membrane potential and firing frequency were averaged in baseline and in a 1-min range containing the point with the maximal change in resting membrane potential after lorcaserin puff. A neuron was considered activated if a change in membrane potential was at least 2 mV, whereas values between a 2 mV were defined as ‘non-responsive’. Clampfit v.10.6 was used to analyze electrophysiology data.

### Resident-intruder test

We used the resident-intruder paradigm to measure social behaviors of male WT and *Htr2c*^F327L/Y^ littermates (4–5 months old) or in female WT and *Htr2c*^F327L/+^ littermates (4 months old) in a semi-natural setting^7^. Mice were singly housed in the resident cage for at least 1 week before testing. The cage remained uncleaned and unchanged for 1 week before testing so that there were olfactory cues to enhance the resident mouse’s territoriality. We started the test by introducing an unfamiliar retired male breeder into the home cage in the afternoon and 7 min later, the intruder was removed from the home cage. All behaviors of mice were continuously recorded with a video camera during the 7-min period. The recorded videos were analyzed in a blinded fashion to measure the time spent by the resident mouse in various behaviors listed below: social exploration (nose–nose sniffing and anogenital sniffing), non-social exploration (self-grooming and cage exploration), defensive behaviors (move away from the intruder, flight and freeze) and offensive behaviors (lateral threat, upright position, clinch attack, keep down and chasing).

### Three-chamber social interaction test

This test was used to evaluate social behavior in male WT and *Htr2c*^F327L/Y^ littermates (4–5 months old) or in female WT and *Htr2c*^F327L/+^ littermates (4 months old). The social interaction test used a three-chambered box with openings between chambers for the mouse to pass through. The test had three sessions: habituation, sociability and social novelty. During the habituation, we put an empty pencil cup upside down into each of the side chambers and a test mouse was released into the center chamber and allowed to explore all the chambers for 15 min. After the habituation session, one novel object was put into the pencil cup on one side and a never-before-met intruder mouse (mouse 1) was placed under the cup on the other side. The sociability session took 15 min. A second never-before-met intruder mouse (mouse 2) was used during the social novelty session to swap out the novel object under the pencil cup. The test mouse again had 15 min to investigate each chamber. The time spent sniffing each pencil cup was recorded. During the sociability session, we calculated the ratio between time spent sniffing mouse 1 and total time spent sniffing mouse 1 or the object to reflect test mouse’s sociability; in the social novelty session, we calculated the ratio between time spent sniffing mouse 2 and total time spent sniffing mouse 1 or mouse 2 to reflect test mouse’s social novelty.

### Risk assessment in the elevated plus-maze test

The EPM apparatus is a ‘+’ shaped maze made of plexi-glass. It consists of two open arms, two closed arms and a center area that are elevated above the floor. The test starts once the animal is placed into the center area. As the animal freely explores the whole maze areas, its behavior is recorded by a camera mounted above the maze. The test lasts for 6 min. After the test, videos were analyzed using Noldus EthoVision XT (v.14.0) or by one tester for different behaviors. The number of visits to open arms and the time spent in open arms were calculated. One tester was blind to the group information of each mouse and counted other behaviors in the videos. When a mouse stands at the center area deciding which arm to enter, the mouse will show a stretch-attend posture with head and shoulders stretching toward the open arms. This posture is defined at ‘risk assessment behavior’. The tester counted the number and the duration of risk assessment behavior in the boundary between the center area and the open arms. When the mouse explores the open arm, the mouse sometimes will show a head dipping posture standing at the side edge of the open arms with head and shoulders dipping down toward the floor. The number and duration of head dipping behavior was counted by the tester as an indication of fearless behavior.

### Statistical analysis

All results were analyzed using GraphPad Prism 8 (GraphPad Software) to evaluate normal distribution and variations within and among groups. For inositol triphosphate turnover assays β-arrestin coupling assays, statistical significance of differences in E_max_ and EC_50_ between WT and variant receptors was determined using unpaired Student’s *t*-tests with Welch correction. For all animal studies, the minimal sample size was predetermined by the nature of experiments. For most of physiological readouts (such as body weight, food intake, energy expenditure and body composition), at least six different mice per group were included. For histology studies, the same experiment was repeated in at least three different mice. For electrophysiological studies, at least 30 different neurons from three different mice were included. The data are presented as mean ± s.e.m. or as individual data points. Methods of statistical analyses were chosen based on the design of each experiment and are indicated in figure legends. *P* < 0.05 was considered to be statistically significant.

### Reporting summary

Further information on research design is available in the Nature Portfolio Reporting Summary linked to this article.

## Online content

Any methods, additional references, Nature Portfolio reporting summaries, source data, extended data, supplementary information, acknowledgements, peer review information; details of author contributions and competing interests; and statements of data and code availability are available at 10.1038/s41591-022-02106-5.

## Supplementary information

Reporting Summary

Supplementary Tables 1 and 2 Cell surface expression and signaling properties of 5HT_2C_R mutants. Generic amino acid residues are according to the numbering scheme proposed by GPCRdb (gpcrdb.org). Variants are classified as LOF, WT-like (-) or gain-of-function (GOF). EC_50_ and maximum response (5-HT efficacy) measured for a given variant relative to WT (E_max_); inositol monophosphate (IP-1); half-maximal inhibitory concentration of 5-HT (IC_50_); relative number of binding sites compared to WT (B_max_); ratio between EC_50_ or IC_50_ measured for a given variant compared to WT (Fmut). Differences between WT and mutant receptors were compared using two-tailed Student’s *t*-test with Welch correction; *P < 0.05, **P < 0.01, *** *P* < 0.001, NS, not significant. Phenotypes in carriers of *HTR2C* variants in the UK Biobank. UK Biobank carriers of *HTR2C* variants found in people with severe early-onset obesity and functionally characterized in this study, shown by genotype and availability of Mental Health questionnaire data.

## Data Availability

Exome sequencing data are accessible from the European Genome Archive under a managed access agreement (EGAS00001000124 and EGAS00001000825). Source data from cell based and animal studies are provided. Anonymized clinical data are available to bonafide researchers from the corresponding author with no restrictions (I.S.F.). Source data are provided with this paper.

## Source data

Source Data Fig. 3 Statistical Source Data.
Source Data Fig. 4 Statistical Source Data.
Source Data Fig. 5 Statistical Source Data.
Source Data Extended Data Fig. 2 Statistical Source Data.
Source Data Extended Data Fig. 3 Statistical Source Data.
Source Data Extended Data Fig. 4 Statistical Source Data.
Source Data Extended Data Fig. 5 Statistical Source Data.
Source Data Extended Data Fig. 6 Immunofluorescence images.
Source Data Extended Data Fig. 7 Statistical Source Data.
