# Supplementary material for: Human loss-of-function variants in the serotonin 2C receptor associated with obesity and maladaptive behavior
Source: Nat Med. 2022 Dec 19;28(12):2537–46. doi: 10.1038/s41591-022-02106-5 (PMC9800280; doi:10.1038/s41591-022-02106-5)
Supplement: Supplementary file 1 — Reporting Summary [file 41591_2022_2106_MOESM1_ESM.pdf]

nature portfolio

Corresponding author(s): Professor I. Sadaf Farooqi
Professor Yong Xu

Last updated by author(s): Oct 18, 2022

Reporting Summary

Nature Portfolio wishes to improve the reproducibility of the work that we publish. This form provides structure for consistency and transparency in reporting. For further information on Nature Portfolio policies, see our Editorial Policies and the Editorial Policy Checklist.

Statistics

For all statistical analyses, confirm that the following items are present in the figure legend, table legend, main text, or Methods section.

n/a	Confirmed	
☐	☒	The exact sample size (*n*) for each experimental group/condition, given as a discrete number and unit of measurement
☐	☒	A statement on whether measurements were taken from distinct samples or whether the same sample was measured repeatedly
☐	☒	The statistical test(s) used AND whether they are one- or two-sided<br>*Only common tests should be described solely by name; describe more complex techniques in the Methods section.*
☐	☒	A description of all covariates tested
☐	☒	A description of any assumptions or corrections, such as tests of normality and adjustment for multiple comparisons
☐	☒	A full description of the statistical parameters including central tendency (e.g. means) or other basic estimates (e.g. regression coefficient) AND variation (e.g. standard deviation) or associated estimates of uncertainty (e.g. confidence intervals)
☐	☒	For null hypothesis testing, the test statistic (e.g. *F*, *t*, *r*) with confidence intervals, effect sizes, degrees of freedom and *P* value noted<br>*Give P values as exact values whenever suitable.*
☒	☐	For Bayesian analysis, information on the choice of priors and Markov chain Monte Carlo settings
☒	☐	For hierarchical and complex designs, identification of the appropriate level for tests and full reporting of outcomes
☒	☐	Estimates of effect sizes (e.g. Cohen's *d*, Pearson's *r*), indicating how they were calculated

Our web collection on statistics for biologists contains articles on many of the points above.

Software and code

Policy information about availability of computer code

Data collection	The pClamp 10.3 software was used to collect electrophysiology results. TSE PhenoMaster metabolic cages were used to collect the data of food intake (48 hr), energy expenditure (02 consumption, CO2 production and heat production) and physical activity (XV axis and Z axis movements).
Data analysis	Graph Pad Prism version 8. The ImageJ was used to analyze Western Blotting and fluorescent microscopic results. We used Harmony software version 4.9.2137.273, revision: 147881 for fluorescent microscopic results. Clampfit version 10.6 was used to analyze electrophysiology data. Noldus EthoVision XT software version 14.0 was used to analyze video recordings from Elevated Plus Maze test.

For manuscripts utilizing custom algorithms or software that are central to the research but not yet described in published literature, software must be made available to editors and reviewers. We strongly encourage code deposition in a community repository (e.g. GitHub). See the Nature Portfolio guidelines for submitting code & software for further information.

Data

Policy information about availability of data

All manuscripts must include a data availability statement. This statement should provide the following information, where applicable:

- Accession codes, unique identifiers, or web links for publicly available datasets
- A description of any restrictions on data availability
- For clinical datasets or third party data, please ensure that the statement adheres to our policy

GOOS WES data are accessible from the European Genome-phenome Archive-EGA:EGAS00001000124. Access to the UK Biobank genotype and phenotype data are open to all approved health researchers, accessible through https://www.ukbiobank.ac.uk. Requests for anonymised human data may be addressed to the corresponding author (I.S.F.) - limitations on clinical data (which has to be anonymised) are designed to protect and respect patient and participant confidentiality.

Molecular data for functional experiments has been submitted as source data.

Field-specific reporting

Please select the one below that is the best fit for your research. If you are not sure, read the appropriate sections before making your selection.

☒ Life sciences ☐ Behavioural & social sciences ☐ Ecological, evolutionary & environmental sciences

For a reference copy of the document with all sections, see nature.com/documents/nr-reporting-summary-flat.pdf

Life sciences study design

All studies must disclose on these points even when the disclosure is negative.

Sample size	In this study, we sought to determine the clinical phenotype associated with rare genetic mutations in HTR2C. For this reason, all participants harbouring rare variants were included. In the functional study, sample size was determined based on similar studies in this field (Lotta, et al., 2019)
Data exclusions	No data was excluded.
Replication	Rare variants identified by exome sequencing were validated by Sanger sequencing. All mutations were validated using this method.<br>In functional studies, reported results were consistently replicated across multiple experiments. All experiments were repeated at least three times.
Randomization	This was an observational and mechanistic study, therefore randomization was not required.
Blinding	For human data collection, investigators were not blinded. Blinding during collection was not needed because conditions were well controlled. Blinding is also not necessary because the results are quantitative and did not require subjective judgment or interpretation.<br><br>For animal bahavioral studies, the experimenter who conducted and analyzed the data was blinded to the genotype and grouping of the animals.

Reporting for specific materials, systems and methods

We require information from authors about some types of materials, experimental systems and methods used in many studies. Here, indicate whether each material, system or method listed is relevant to your study. If you are not sure if a list item applies to your research, read the appropriate section before selecting a response.

Materials & experimental systems

n/a	Involved in the study	
☐	☒	Antibodies
☐	☒	Eukaryotic cell lines
☒	☐	Palaeontology and archaeology
☐	☒	Animals and other organisms
☐	☒	Human research participants
☒	☐	Clinical data
☒	☐	Dual use research of concern

Methods

n/a	Involved in the study	
☒	☐	ChIP-seq
☒	☐	Flow cytometry
☒	☐	MRI-based neuroimaging

Antibodies

Antibodies used	Supplier name, Catalog number, clone name, lot number.<br>Mouse anti-FLAG M2 antibody (dilution 1:1000) (F1804, Sigma-Aldrich)<br>Goat anti-mouse IgG(H+L)-HRP conjugate (dilution 1:1250) (172-1011, Bio-Rad Laboratories)<br>Rabbit polyclonal anti-calreticulin antibody (dilution 1:100) (PA3-900, Invitrogen)<br>Rabbit polyclonal anti-β-endorphin (dilution 1:10000) (#H-022-33, Phoenix Peptide)<br>Mouse monoclonal anti-c-Fos (dilution 1:1000) (Ab208942, Abcam)<br>Alexa Fluor™ 488 goat anti-mouse IgG (H+L) (dilution 1:400) (A11029, Life Technologies)<br>Alexa Fluor™ 647 donkey anti-rabbit IgG (H+L) (dilution 1:400) (A31573, Thermo)<br>Alexa Fluor™ 488 donkey anti-rabbit IgG (H+L) (dilution 1:200) (A21206, Invitrogen)<br>AlexaFluor™ 594 donkey anti-mouse IgG (H+L) (dilution 1:200) (A21203, Invitrogen)
Validation	The anti-β-endorphin (#H-022-33, Phoenix Peptide) has been validated in several previous work (listed in the website) and validated in our previous work (He et al., 2021). Anti-c-Fos (Ab208942, Abcam) antibody has been validated by Abcam (https://www.abcam.com/c-fos-antibody-2h2-ab208942.html) and also validated in our previous work (He et al., 2021).<br>Anti-FLAG is standard commercially available antibody and validated by Sigma-Aldrich (https://www.sigmaaldrich.com/GB/en/

product/sigma/f1804) and also used previously (Lotta, et al., 2019). Anti-calreticulin antibody was validated by Invitrogen (https://www.thermofisher.com/antibody/product/Calreticulin-Antibody-Polyclonal/PA3-900)

Eukaryotic cell lines

Policy information about cell lines

Cell line source(s)	HEK293 cells were kindly provided by Professor Dario Alessi (MRC Protein Phosphorylation and Ubiquitylation Unit, University of Dundee). HEK293SL cells were kindly provided by Professor Michel Bouvier, Université de Montréal.
Authentication	HEK293 cells were authenticated via GENETICA Genotypes Analysis in May 2019, showing 97% match when compared to the reference profile ATCC sequence.
Mycoplasma contamination	HEK293 cells were tested negative for mycoplasma contamination using MycoProbe Mycoplasma Detection Kit (CUL001B, R&D Systems).
Commonly misidentified lines (See ICLAC register)	None

Animals and other organisms

Policy information about studies involving animals; ARRIVE guidelines recommended for reporting animal research

Laboratory animals	We used laboratory mice as the model system. All study mice are at a C57Bl6J background. The age and sex of mice used in each study were clearly described in the figure and figure legends.
Wild animals	No wild animals were used in the study.
Field-collected samples	No field collected samples were used in the study.
Ethics oversight	Care of all animals and procedures were approved by the Baylor College of Medicine Institutional Animal Care and Use Committee.

Note that full information on the approval of the study protocol must also be provided in the manuscript.

Human research participants

Policy information about studies involving human research participants

Population characteristics	Participants recruited from the Genetics of Obesity Study (www.goos.org.uk) were referred by their physicians based on the following criteria:<br>(1) Early onset obesity (before age 10 years)<br>(2) Severe obesity as defined by BMI ≥ 3 standard deviation scores.<br>All participants found to have missense mutations in HTR2C and their family members were invited to participate. Sex was assigned by clinical examination by Physicians.
Recruitment	Participants were referred to the Genetics of Obesity Studies by their physicians if they satisfied the criteria listed above, irrespective of their physical location. The travel costs of participants were reimbursed. No other reimbursement for taking part was provided.
Ethics oversight	All studies were approved by the Multi-Regional Ethics Committee and the Cambridge Local Research Ethics Committee (MREC 97/21 and REC number 03/103). All participants, or their legal guardian for those aged under 16, provided written consent for all assessments; in addition, participants under the age of 16 provided oral assent.

Note that full information on the approval of the study protocol must also be provided in the manuscript.
