# Supplementary material for: Human loss-of-function variants in the serotonin 2C receptor associated with obesity and maladaptive behavior
Source: Nat Med. 2022 Dec 19;28(12):2537–46. doi: 10.1038/s41591-022-02106-5 (PMC9800280; doi:10.1038/s41591-022-02106-5)
Supplement: Source Data Extended Data Fig. 6 — Immunofluorescence images. [file 41591_2022_2106_MOESM10_ESM.pptx]

## Slide 1
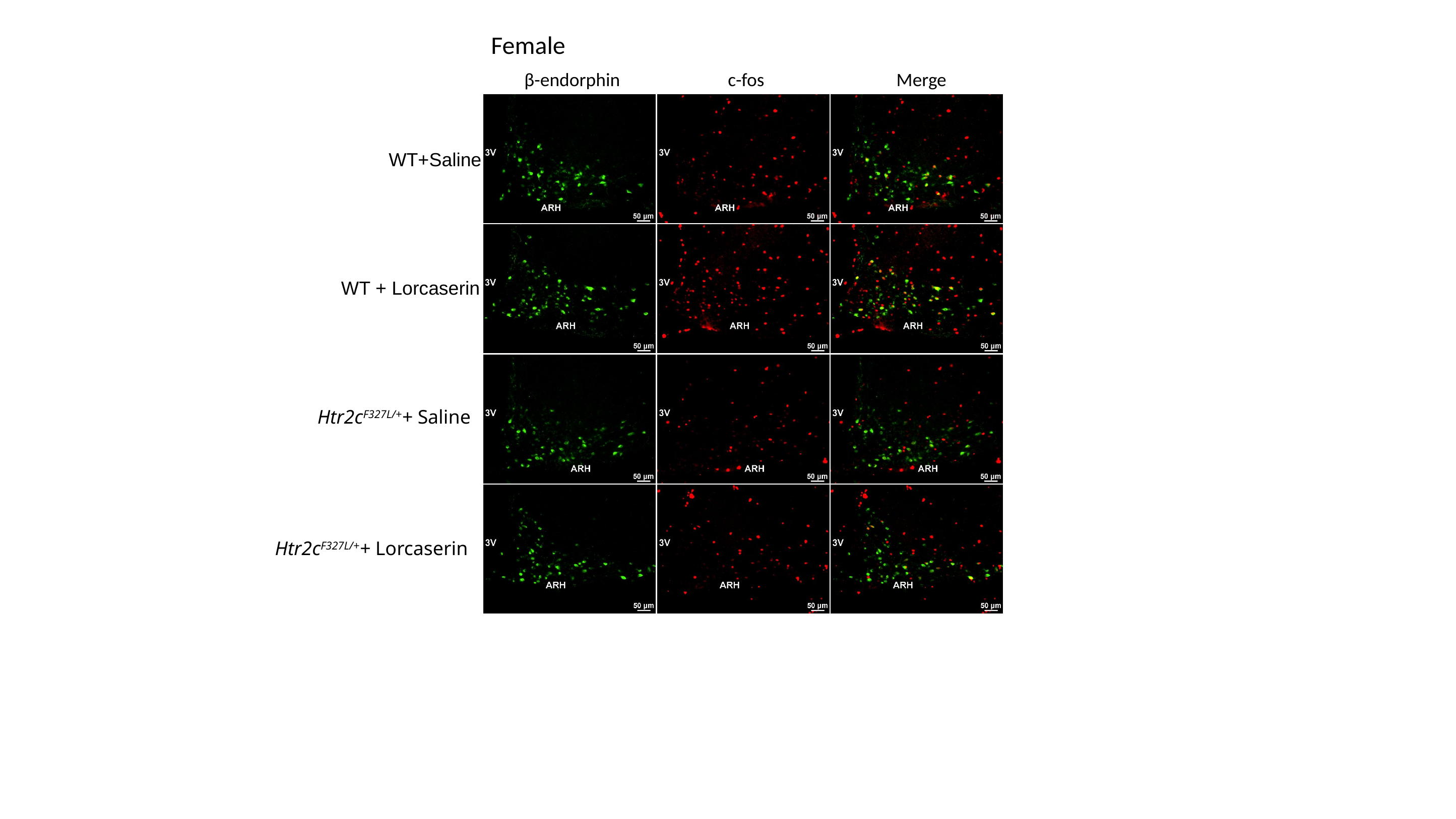

Female
β-endorphin
c-fos
Merge
WT+Saline
WT + Lorcaserin
Htr2cF327L/++ Saline
Htr2cF327L/++ Lorcaserin

## Slide 2
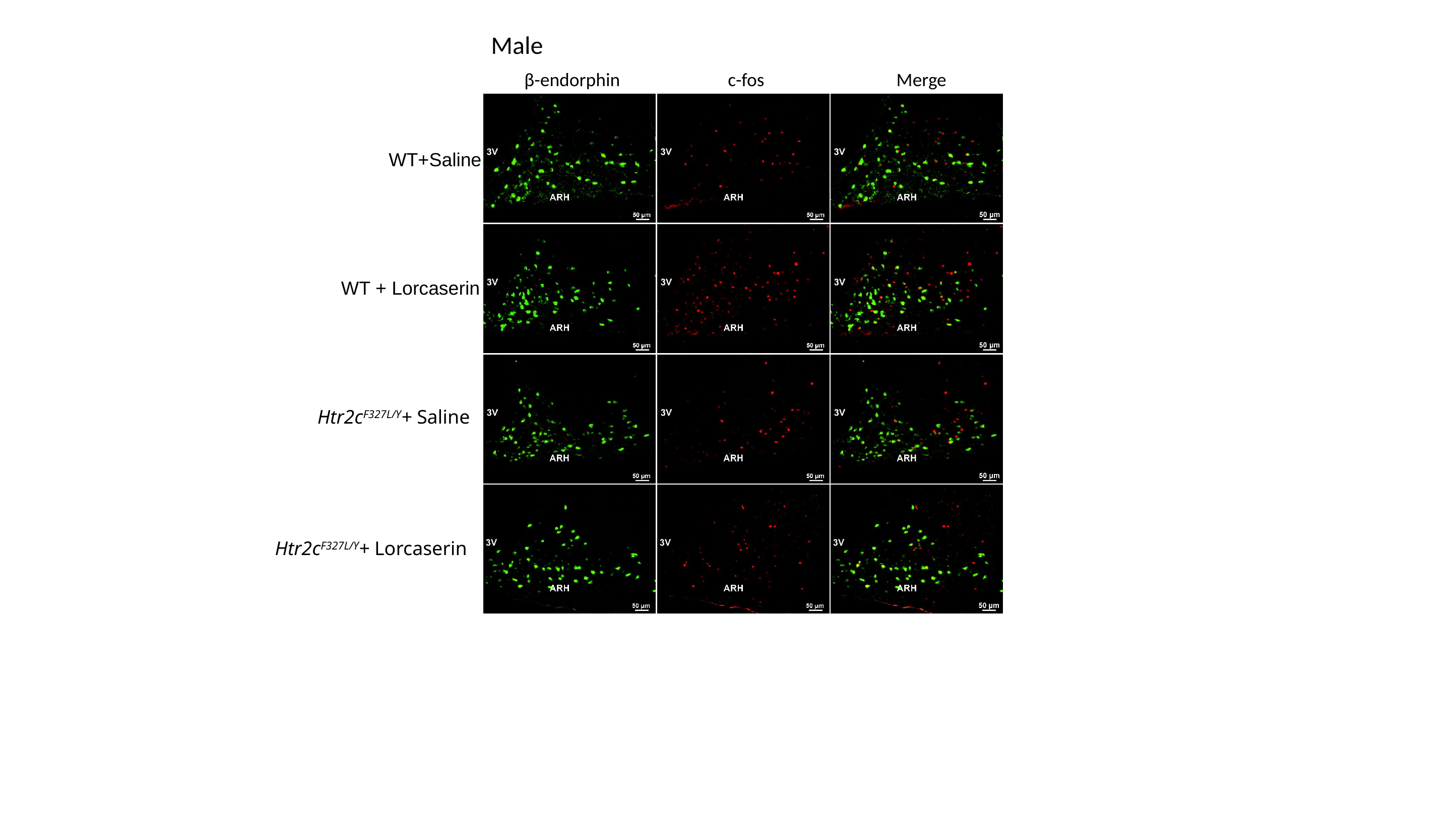

Male
β-endorphin
c-fos
Merge
WT+Saline
WT + Lorcaserin
Htr2cF327L/Y+ Saline
Htr2cF327L/Y+ Lorcaserin
